# LDB1 represses fetal hemoglobin expression by enhancing *BCL11A* transcription

**DOI:** 10.1016/j.redox.2026.104070

**Published:** 2026-02-04

**Authors:** Si-Won Park, Chang-Yong Choi, In-Byung Park, Seok-Jin Kang, Hyeon Jeong Lee, Ji-In Kim, Joonbeom Bae, Dongho Geum, Yong-Pil Cheon, Taehoon Chun

**Affiliations:** a Department of Biotechnology, College of Life Sciences and Biotechnology, Korea University, Seoul, 02841, Republic of Korea; b Department of Biomedical Sciences, College of Medicine, Korea University, Seoul, 02841, Republic of Korea; c Division of Developmental Biology and Physiology, Department of Biotechnology, Institute for Basic Sciences, Sungshin University, Seoul, 02844, Republic of Korea

**Keywords:** BCL11A, Fetal globin regulation, LDB1, Hemoglobin switching, Reactive oxygen species

## Abstract

Deciphering the mechanism governing the temporal switch from fetal to adult hemoglobin during erythropoiesis has significant clinical relevance. Here, we identify LDB1 as a pivotal regulator of β-globin switching in erythroid progenitors. The absence of LDB1 in proerythroblasts from mouse fetal liver leads to cell cycle arrest and apoptosis due to the accumulation of reactive oxygen species (ROS), resulting from excessive heme content caused by significant overexpression of embryonic β-globin genes such as *Hbb-y* and *Hbb-bh1*. Mechanistically, LDB1 directly enhances the mRNA expression of fetal globin gene repressors, including *Bcl11a*, *Cbfa2t3*, and *Sox6*. Moreover, the LDB1 complex, which includes LMO2 and GATA1, binds directly to enhancer regions of *Bcl11a*, promoting its transcription. CRISPR/Cas9-mediated LDB1 knockout in human erythroleukemia cells confirmed LDB1 as a key enhancer of *BCL11A* transcription, reducing its mRNA expression while upregulating transcription of the fetal globin gene *HBG*. Following chromatin immunoprecipitation (ChIP) assays revealed LDB1 binding to intron 2 enhancers within the *BCL11A* locus, reinforcing its indispensable role in *BCL11A* transcription in humans. Consequently, ectopic expression of BCL11A in LDB1-deficient proerythroblasts promotes their proliferation by rescuing them from ROS-mediated apoptosis. These findings highlight the essential role of LDB1 in fetal globin silencing during erythropoiesis.

## Highlights

• LDB1 deficiency in proerythroblasts causes ROS-mediated apoptosis by excessive heme.
• LDB1 regulates fetal globin to prevent ROS-mediated apoptosis in erythroblasts.
• LDB1 transcriptionally activates BCL11A, fetal β-globin repressor.
• The LDB1 complex binds BCL11A enhancers to promote its transcription.

## Graphical abstract

**Figure:**
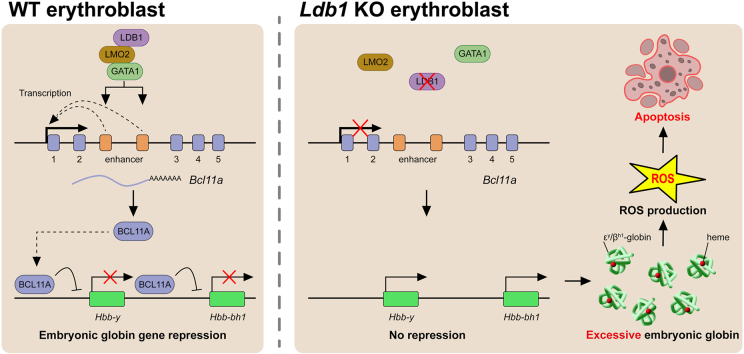

## 1 Introduction

Erythropoiesis is a meticulously controlled process that guides precursor cells through multiple stages of proliferation and differentiation, ultimately producing mature erythrocytes [1,2]. During mammalian embryogenesis, two distinct erythroid lineages give rise to terminally differentiated erythrocytes [1,2]. The precursor cells of the primitive (embryonic) erythroid lineage are first visualized on embryonic day 7.5 (E7.5) in the mouse yolk sac, forming primitive erythroid cells (EryPs) [1,2]. Colony-forming cells with high proliferative potential (HPP–CFCs), which are the initial precursors of the definitive (adult) erythroid lineage, appear in the mouse yolk sac by E8.5 and begin producing definitive erythroid cells (EryDs) [3]. After E12.5, hematopoietic stem cells (HSCs) in the fetal liver (FL) of mice take over the function of HPP-CFCs and continue to generate EryDs [1,2]. Postnatally, HSCs in the bone marrow (BM) maintain the production of EryDs [1,2].

One of the critical steps during erythropoiesis is hemoglobin switching, a transition that is vital for effective oxygen delivery postnatally [4,5]. In mice, the expression pattern of embryonic β-globin genes such as *Hbb-y* and *Hbb-bh1* is limited to EryPs, while adult β-globin genes such as *Hbb-b1* and *Hbb-b2* are exclusively expressed in EryDs starting at E12.5 in the FL [4,5]. In humans, the transition from embryonic ε-globin to fetal γ-globin occurs as erythropoiesis moves from the yolk sac to the FL [4,5]. Following this transition, EryDs predominantly produce γ-globin, which combines with α-globin to produce fetal hemoglobin (HbF) [4,5]. After birth, γ-globin expression decreases, while β-globin expression increases, resulting in the formation of adult hemoglobin (HbA) [4,5]. Failure of the developmental switch from γ-globin to β-globin, due to dysregulated globin gene expression, causes hematological disorders such as β-thalassemia, in which the accumulation of unpaired α-globin chains induces reactive oxygen species (ROS)-mediated apoptosis in red blood cells (RBCs) [6,7].

LDB1, which forms a multiprotein complex with another nuclear adaptor protein LMO2 and transcription factors such as GATA1 and TAL1, is considered a critical upstream component of erythropoiesis through long-range promoter-enhancer interactions [8]. Indeed, genome-wide mapping of LDB1 binding sites using mouse erythroleukemic (MEL) cells or mouse primary bone marrow cells (BMCs) has revealed that LDB1 complexes directly enhance the expression of numerous erythroid genes involved in various stages of erythropoiesis, including β-globin genes [9,10]. Consistent with this notion, *Ldb1*-deficient mouse yolk sacs are incapable of sustaining primitive erythropoiesis [11,12]. Moreover, mice harboring a conditional deletion of *Ldb1* using Tie2cre and Mx1cre exhibit defects in the long-term HSCs (LT-HSCs), megakaryocyte-erythroid progenitors (MEPs), megakaryocytes, and erythrocytes [12,13]. These results confirm that LDB1 function is essential for both embryonic and definitive erythropoiesis.

While both conditional knockout models have been valuable for studying functional roles of LDB1 in HSCs and erythropoiesis, they have limitations in examining LDB1's role in definitive erythropoiesis, particularly in the FL. The Tie2cre system can knock out genes as early as E10.5, efficiently deleting target genes in FL HSCs. However, it can also cause endothelial cell-specific deletion, thereby exhibiting extrinsic effects on non-hematopoietic cells [14]. Consequently, all *Ldb1*^fl/fl^Tie2cre ^+^ mouse embryos died at E12.5 due to idiopathic hemorrhage and edema [12]. Therefore, the *Ldb1*^fl/fl^Tie2cre ^+^ mouse could be a useful tool for studying the role of LDB1 in the primitive (embryonic) lineage, but it is not well-suited for studying its role in the definitive (adult) erythroid lineage. In *Ldb1*^fl/fl^Mx1cre ^+^ mice, polyinosinic-polycytidylic acid injection activates type I interferon signaling, followed by transient anemia, which may complicate the interpretation of phenotypes observed in these mice [12,15,16].

In this study, we generated and characterized a conditional deletion of *Ldb1* using the Vav1cre system, which specifically targets HSCs, to investigate LDB1's role in definitive erythropoiesis in the FL without the confounding effects observed in Tie2cre or Mx1cre systems [17]. Unlike the phenotype of *Ldb1*^fl/fl^Tie2cre ^+^ mice, *Ldb1*^fl/fl^Vav1cre^+^ (KO) mice did not exhibit embryonic lethality. However, the KO mice survived less than 5 days after birth due to defective definitive erythropoiesis. Surprisingly, we observed a significant accumulation of MEPs, most of which were proerythroblasts, in E14.5 KO FLs compared to those in *Ldb1*^fl/fl^Vav1cre^−^ (WT) mice. Subsequent transcriptional, epigenomic, and promoter binding analyses revealed that LDB1 directly enhances the mRNA transcription of fetal globin gene repressors such as *Bcl11a*, *Cbfa2t3* (*Eto2*), and *Sox6*. Consequently, *Ldb1* deficiency causes significant overexpression of embryonic globin genes such as *Hbb-y* and *Hbb-bh1*, which is accompanied by increased heme content in KO proerythroblasts. This is further associated with elevated ROS levels and increased apoptosis in KO proerythroblasts. Conversely, the ectopic expression of BCL11A in KO proerythroblasts significantly alleviates the defective phenotypes. Furthermore, CRISPR/Cas9-induced LDB1 knockout in human erythroleukemia cells confirmed that LDB1 functions as an important regulator of *BCL11A* transcription, decreasing its mRNA expression while increasing the mRNA expression of the fetal globin gene *HBG*. Subsequent chromatin immunoprecipitation (ChIP) assays demonstrated LDB1 binding to enhancers in intron 2 of the *BCL11A* locus, further supporting its essential role in the transcriptional activation of *BCL11A* in humans. These results provide the first evidence that LDB1 is directly involved in fetal globin silencing during erythropoiesis. Therefore, LDB1 acts as an important upstream component of the globin switching network, potentially facilitating adult globin gene expression while limiting fetal globin gene transcription.

## 2 Material and methods

### 2.1 Experimental mice and phenotypic analysis

The KO and the WT mice were generated by crossing *Ldb1*^fl/fl^ mice [18] with Vav1cre mice (The Jackson Laboratory) and then maintained on the C57BL/6 (The Jackson Laboratory) background after a minimum of 10 backcrosses. CD45.1 mice on a C57BL/6 background were obtained from The Jackson Laboratory for the competitive repopulation assay. The primer pairs used for genotyping of the WT and KO mice are detailed in Supplementary Table S1. The lymphoid tissues of the WT and KO mice at E14.5, E16.5, postnatal day 1 (P1), and P3 were isolated to analyze each hematopoietic lineage cell population using flow cytometry. All animals received proper care in accordance with the National Institutes of Health Guide for the Care and Use of Laboratory Animals. The study protocol was approved by the Institutional Animal Care and Use Committee of Korea University (protocol numbers: KUIACUC-2019-0011 and KUIACUC-2022-0046).

### 2.2 Flow cytometry analysis and cell sorting

**Fig. 1:**
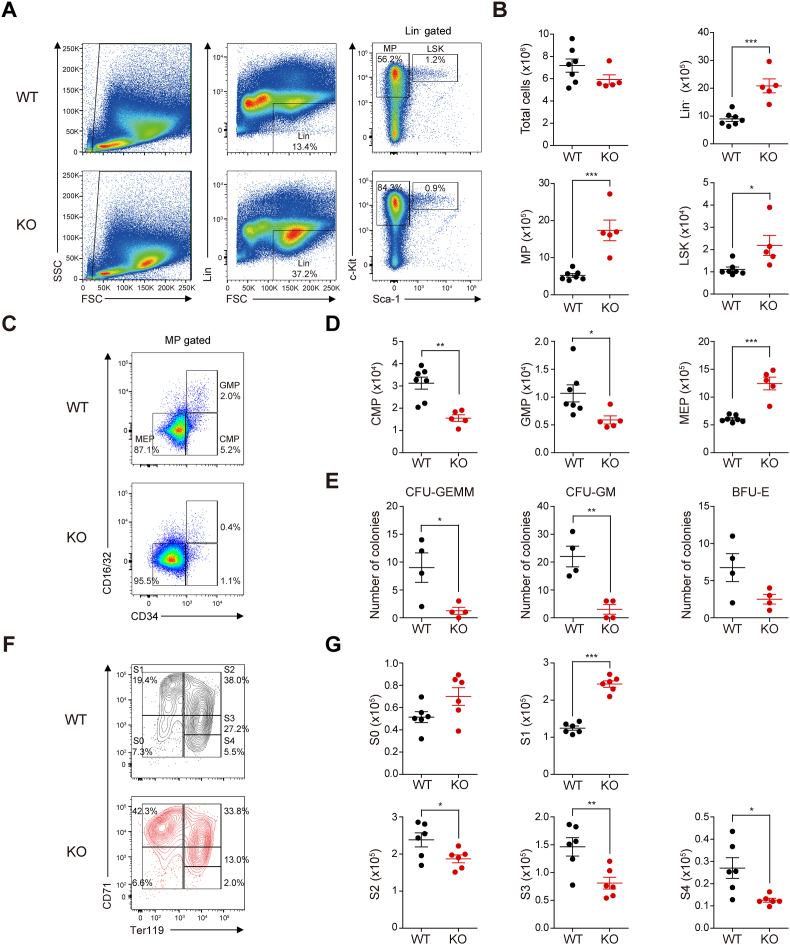
Hematopoietic cell-specific *Ldb1* deletion leads to an accumulation of erythroid progenitors, impairing their progression to fully mature erythrocytes. (**A** and **B**) Representative flow cytometry plots (**A**), and absolute numbers of total cells, Lin^−^ cells, myeloid progenitors and LSK (Lin^−^Sca-1^+^c-kit^+^) cells (**B**) from E14.5 WT (*n* = 7) and KO (*n* = 5) FLs. (**C** and **D**) Representative flow cytometry plots (**C**), and absolute numbers of common myeloid progenitors (CMP), granulocyte-monocyte progenitors (GMP) and megakaryocyte-erythroid progenitors (MEP) (**D**) from E14.5 WT (*n* = 7) and KO (*n* = 5) FLs. (**E**) Clonogenic progenitors from E14.5 WT and KO FLCs were assessed by CFU assays. *n* = 4. CFU-GEMM, CFU-granulocyte/erythrocyte/monocyte/megakaryocyte; CFU-GM, CFU-granulocyte and/or macrophage; BFU-E, erythroid burst-forming units. (**F** and **G**) Representative flow cytometry plots (**F**), and absolute numbers of erythrocyte precursors (**G**) from E14.5 WT and KO FLs. *n* = 6. S0, early erythroid progenitors (Ter119^−^CD71^low/med^ cells, S0 cells); S1, proerythroblasts (Ter119^−^CD71^high^ cells, S1 cells); S2, basophilic erythroblasts (Ter119^+^CD71^high^ cells, S2 cells); S3, late basophilic and polychromatophilic erythroblasts (Ter119^+^CD71^med^ cells, S3 cells); S4, orthochromatophilic erythroblasts (Ter119^+^CD71^low^ cells, S4 cells). Statistical significance was assessed by two-tailed Student's *t*-test. ∗*P* < 0.05; ∗∗*P* < 0.01; ∗∗∗*P* < 0.001. All data are presented as the mean ± SEM.

Single cell suspensions were prepared from the lymphoid tissues of each mouse or fetus for flow cytometry. After removing erythrocytes using RBC lysis buffer (Biolegend 420301), the cells were preincubated with Fc Block (BD Biosciences 553141, dilution 1:100) in staining buffer (2% FBS and 2 mM EDTA in PBS) at 4 °C for 30 min. After washing with PBS, the cells were incubated with the appropriate antibodies (Abs) in staining buffer at 4 °C for 30 min [19]. Abs purchased from BD Biosciences, Biolegend or Thermo Fisher Scientific were employed to detect the following molecules by flow cytometry: B220 (BD Biosciences 553090, dilution 1:100), CD16/32 (Thermo Fisher Scientific 45-0161-82 or 25-0161-82, dilution 1:100), CD34 (BD Biosciences 553733, dilution 1:100), CD41 (BD Biosciences 553848, dilution 1:100), CD71 (Thermo Fisher Scientific 12-0711-82, dilution 1:100), CD105 (BD Biosciences 564744, dilution 1:100), CD127 (IL-7Rα) (Thermo Fisher Scientific 45-1271-80, dilution 1:100), CD150 (SLAM) (Biolegend 115921, dilution 1:100), c-kit (Thermo Fisher Scientific 17-1171-81, dilution 1:100), Gr-1 (Ly-6G/Ly-6C) (Thermo Fisher Scientific 11-5931-82, dilution 1:100), Sca-1 (Ly-6A/E) (BD biosciences 553108 or 558162, dilution 1:100), streptavidin (BD Biosciences 554060 or 554063, dilution 1:100), and Ter119 (Thermo Fisher Scientific 11-5921-82, 17-5921-82 or 25-5921-82, dilution 1:100). To measure expression of BCL11A, the cells were stained with anti-BCL11A Ab (Santa Cruz Biotechnology sc-514842 AF488, dilution 1:100) in intracellular staining buffer (Thermo Fisher Scientific 00-5523-00). After washing several times with PBS, stained cells were resuspended in PBS and analyzed by flow cytometry using a FACSVerse™ with FACSuite software (BD Biosciences). Depletion of specific lineage cells was achieved by lineage cell depletion kit (Miltenyi Biotec 130-090-858) with a magnetic associated cell separation (MACS) system (Miltenyi Biotec) according to the manufacturer's protocol. Common myeloid progenitors (CMPs), granulocyte/macrophage progenitors (GMPs), and MEPs were isolated from myeloid progenitors (Lin^−^Sca-1^−^c-kit^+^ cells) [20] by sorting with a FACSAria II cell sorter (BD Biosciences). The gating strategy for sorting these cells is shown in Fig. 1C. Erythrocyte precursors at each stage (S0 to S4 cells) were also isolated using the FACSAria II sorter, with the gating strategy shown in Fig. 1F.

To measure expression levels of human embryonic ε-globin (HBE1) and fetal hemoglobin (HbF), as well as mouse embryonic ε-globin (HBBY), intracellular staining was performed as described above. For detection of human HBE1 and mouse HBBY, cells were first stained with anti-embryonic ε-globin Ab (Thermo Fisher Scientific 12361-1-AP, dilution 1:98.25) or its isotype control (Proteintech 30000-0-AP, dilution 1:250), followed by incubation with donkey anti-rabbit IgG Ab (Abcam ab150075, dilution 1:2000) as the secondary Ab. For detection of human HbF, cells were stained with anti-fetal hemoglobin Ab (Thermo Fisher Scientific MHFH05, dilution 1:100) or its isotype control (Thermo Fisher Scientific 17-4714-82, dilution 1:200).

### 2.3 Colony forming unit (CFU) assay

Isolated CMPs (5 × 10^4^ cells/well), GMPs (2.5 × 10^4^ cells/well), and MEPs (5 × 10^4^ cells/well) were plated onto Methocult GF M3434 (StemCell Technologies 03444) in 35 mm cell culture dishes. The cells were then incubated at 37 °C in 5% CO_2_. After 7 days of incubation, the type and number of colonies were determined [19].

### 2.4 Competitive repopulation assay

A total of 2 × 10^6^ E14.5 fetal liver cells (FLCs) from the WT or KO fetuses (CD45.2) mixed with 2.5 × 10^5^ BMCs (CD45.1) from 8-week-old WT mice were intravenously injected into lethally irradiated (10 Gy) 8-week-old WT recipient mice (CD45.1) to track donor reconstitution in BM (Supplementary Fig. S3A) [21,22]. Twelve weeks after transplantation, the BMCs of recipient mice (CD45.1) were analyzed to assess donor hematopoietic stem and progenitor cell (HSPC) reconstitution (CD45.2). To distinguish between donor and recipient HSPCs, cells were stained with PE-Cy7 anti-CD45.1 Ab (Thermo Fisher Scientific 25-0453-82, dilution 1:100) or PerCP-Cy5.5 anti-CD45.2 Ab (BD Biosciences 552950, dilution 1:100). The gating strategies for Lin^−^ cells, LSK cells, myeloid progenitors, CMPs, GMPs, and MEPs are shown in Fig. 1. Common lymphoid progenitors were gated as Lin^−^IL-7Ra^+^c-kit^low^Sca-1^low^ cells, granulocytes as Gr-1^+^ cells, and B cells as B220^+^ cells [19]. The Abs used for the analyses are listed above.

### 2.5 Cell cycle analysis

BrdU Flow kits (BD Biosciences 559619 or 552598) were used to analyze the cell cycle of erythrocyte precursors at each stage (S0 to S4 cells) according to the manufacturer's instructions. Briefly, E14.5 WT or KO FLCs were labeled with BrdU in RPMI-1640 (Welgene LM011-01) supplemented with 10% FBS, 20 mM HEPES (Welgene BB001-01), and 1% penicillin/streptomycin (Welgene LS202-02) for 2 h at 37 °C with 5% CO_2_. The cells were harvested and stained with the appropriate Abs listed above or with those provided in the BrdU Flow kits. After several washes with PBS, the stained cells were resuspended in PBS and analyzed by flow cytometry using a FACSVerse™ with FACSuite software (BD Biosciences).

### 2.6 Giemsa staining

E14.5 WT or KO FLCs (2 × 10^5^ cells) were centrifuged onto Superfrost™ plus microscope slides (Thermo Fisher Scientific 12-550-15) using a Shandon Cytospin 4 Cytocentrifuge (Thermo Fisher Scientific). Erythroid cells were stained with StainRITE Giemsa dye (Polysciences 25038-100) following the manufacturer's instructions. After staining, they were characterized under a microscope.

### 2.7 Benzidine staining

For benzidine staining [4,23] × 10^5^ FLCs or sorted erythrocyte precursors (S0 to S4 cells) from E14.5 WT or KO fetuses were washed several times with ice-cold PBS and resuspended in PBS to prepare cell suspensions. A benzidine working solution was prepared by mixing 20 μl of hydrogen peroxide solution (Sigma-Aldrich H1009-100 ML) with 4 ml benzidine stock solution [0.2% benzidine dihydrochloride (Sigma-Aldrich T8768-1G) in 3% acetic acid (Sigma-Aldrich 695092-2.5L)]. The cell suspensions were mixed with the benzidine working solution at a 1:1 ratio for staining. After staining, the cells were examined under a microscope, and the optical density of each reaction was then measured at 652 nm using a multimode microplate reader (Hidex 425-301).

### 2.8 Measurement of heme content

Total cellular heme levels were assessed using established procedures [24]. Briefly, sorted erythrocyte precursors were centrifuged at 2,000*g* for 5 min. After discarding the supernatant, 500 μl of 2 M oxalic acid (Sigma-Aldrich 194131-250G) was added to the pellets. The mixture was then vigorously shaken and heated at 100 °C for 30 min. Heme concentration was quantified by measuring fluorescence with a multimode microplate reader (Hidex 425-301) using excitation and emission wavelengths of 400/662 nm. A blank control, consisting of cells treated with oxalic acid but not heated, was included to account for endogenous porphyrins in the cells. Standard curve was calculated based on data from 0 ng to 20 ng of hemin (Sigma-Aldrich 51280-5G) dissolved in DMSO (Sigma-Aldrich D2650-100 ML) with 500 μl of 2 M oxalic acid solution, followed by heating as described above.

### 2.9 Measurement of relative heme synthesis and degradation

To measure relative heme synthesis between WT and KO proerythroblasts, sorted Ter119^-^ cells (S0 and S1) were cultured in the presence of 0.5 mM 5-aminolevulinic acid (Sigma-Aldrich A3785-500 MG) and/or 0.5 mM succinylacetone (Sigma-Aldrich D1415-100 MG) for 1 h [25]. To assess heme degradation, sorted Ter119^-^ cells (S0 and S1) were cultured in the presence of 0.5 mM succinylacetone for 48 h [25]. Cells were then harvested, and heme contents were measured. Relative heme synthesis and degradation between WT and KO proerythroblasts were subsequently calculated.

### 2.10 Measurement of cellular ROS

Cytoplasmic ROS was measured using the CellROX Deep Red Reagent kit (Thermo Fisher Scientific C10422) according to the manufacturer's instructions. Briefly, 1 × 10^6^ E14.5 FL erythrocyte precursors at each stage (S0 to S4 cells) in 6-well plates were incubated with 5 μM CellROX dye in PBS containing 2% FBS for 30 min at 37 °C with 5% CO_2_. After incubation, the cells were harvested and stained with anti-mouse CD71 and anti-mouse Ter119 Abs and analyzed by flow cytometry, as described above.

### 2.11 In vitro erythroid differentiation

*In vitro* erythroid differentiation assays using erythrocyte precursors from E14.5 FLs were performed as previously described [26]. Briefly, Ter119^-^ cells (S0 and S1 cells) from E14.5 FLs were isolated by MACS-based negative selection after staining with a combination of biotin-conjugated anti-Ter119 Ab (BD Biosciences 553672, dilution 1:10) and anti-biotin microbeads (Miltenyi Biotec 130-090-485, dilution 1:5) using LS columns (Miltenyi Biotec 130-042-401). The purified cells were then seeded into fibronectin (Sigma-Aldrich F0895-1 MG) coated 24-well plates at a density of 1 × 10^5^ cells/mL. On the first day, the cells were cultured in IMDM (Welgene LM004-02) containing 15% FBS, 1% penicillin/streptomycin, 1% detoxified bovine serum albumin (GenDEPOT A0100), 200 μg/mL holo-transferrin (Sigma-Aldrich T4132-100 MG), 10 μg/mL recombinant human insulin (Sigma-Aldrich 91077C-100 MG), 2 mM _l_-glutamine (Welgene LS002-01), 100 μΜ 2-mercaptoethanol (Thermo Fisher Scientific 21985-023), and 2 U/mL recombinant human EPO (Thermo Fisher Scientific PHC2054). On the following day, the medium was replaced with IMDM supplemented with 20% FBS, 1% penicillin/streptomycin, 2 mM _l_-glutamine, and 100 μΜ 2-mercaptoethanol. On the indicated days of differentiation, harvested cells were subjected to cell cycle analysis, as described above. To rescue ROS accumulation in KO proerythroblasts, 1 mM or 2.5 mM N-acetylcysteine (NAC) (Sigma-Aldrich A7250-5G) was added to the culture medium [25,27].

The proliferation and apoptosis of erythrocyte precursors at each stage (S0 to S4 cells) were measured 3 days after differentiation. Briefly, cell proliferation was measured using BrdU labeling reagent as described above. Apoptosis was measured by staining the cells with FITC-conjugated Annexin V (BD Biosciences 556547, dilution 1:20) or PerCP-Cy5.5-conjugated Annexin V (Thermo Fisher Scientific 561431, dilution 1:20) at 4 °C for 20 min. For proliferation and apoptosis assays, the cells were additionally stained with anti-mouse CD71 and anti-mouse Ter119 Abs to distinguish erythrocyte precursors at each stage as described above. After several washes with PBS, the stained cells were analyzed by flow cytometry.

### 2.12 Western blot analysis

Immunoblotting was performed as previously described [19]. The following primary Abs were included: anti-LDB1 Ab (Santa Cruz Biotechnology sc-365074, dilution 1:100), anti-BCL11A Ab (Santa Cruz Biotechnology sc-514842, dilution 1:100), and anti-β-actin Ab (Sigma-Aldrich A5441, dilution 1:20,000). Goat anti-mouse IgG-HRP (Santa Cruz Biotechnology sc-2005, dilution 1:2500) was used as the secondary Ab. Immunoreactive bands were visualized using an ECL solution (Thermo Fisher Scientific 34580).

### 2.13 Real-time quantitative reverse transcription PCR (qRT-PCR analysis)

Total RNA was isolated using a miRNeasy kit (Qiagen 1038703) and subjected to cDNA synthesis with a DiaStar™ RT Series kit (SolGent DR23-R10k) according to the manufacturer's protocols. qRT-PCR was performed using SYBR Green master mix (CellSafe QG-05). Following qRT-PCR, the mRNA levels of each gene were quantified using the CFX Connect Real-Time PCR detection system (Bio-Rad). The housekeeping gene *Gapdh* was used as an internal control to normalize the mRNA levels of other genes. The primer pairs used for qRT-PCR are listed in Supplementary Table S1.

### 2.14 RNA-seq analysis

RNA-Seq data analysis was performed as previously described [19]. Briefly, total RNA was extracted from sorted E14.5 FL proerythroblasts (S1 cells) of WT and KO mice using the miRNeasy Mini Kit, following the manufacturer's instructions. The RNA concentration was measured with the Quant-it™ RiboGreen RNA assay kit (Thermo Fisher Scientific R11490), and RNA integrity was assessed by running the samples on a TapeStation RNA ScreenTape (Agilent Technologies 5067-5576). Each RNA library was independently prepared using 1 μg of total RNA per sample with the Illumina TruSeq Stranded mRNA Sample Prep Kit (Illumina RS-122-2101), ensuring that only high-quality RNA samples with a RIN value greater than 7.0 were used. The remaining procedures for RNA-seq analysis, including paired-end sequencing, alignment, and data analysis, are detailed in an earlier publication [19].

### 2.15 ChIP-seq dataset analysis

Raw sequencing reads from a published dataset of the MEL cells [European Nucleotide Archive (ENA) accession: ERA000161] were obtained and analyzed to investigate potential LDB1 binding sites in erythroid cells [9]. Before the analysis, adapter sequences and low-quality bases (score <3) were trimmed using Trimmomatic (version 0.38) [28]. A sliding window filter (4-base window, mean quality score ≥15) was applied, and reads shorter than 36 bp were discarded to ensure data quality. The reference *Mus musculus* genome (mm10) was used to align the cleaned reads with Bowtie (version 1.1.2) [29]. After the alignment process, SAMtools (version 1.9) [30] was utilized to sort and index the mapped data. To ensure data accuracy, duplicate reads were eliminated using the MarkDuplicates module in Picard (version 1.118). The model-based analysis of ChIP-Seq data (MACS2 version 2.1.1) [31] was used to identify peaks in the aligned sequence data. Subsequently, ChIPseeker (version 1.18.0) [32] was employed to annotate the genes and transcripts located near the peaks identified by MACS2.

### 2.16 ChIP-qPCR assay

The lysates of E14.5 FLCs or K562 cells (ATCC CCL-243) were prepared for the ChIP-qPCR assay following established protocols [19]. Briefly, 1 × 10^7^ cells were incubated with 1% formaldehyde (Sigma-Aldrich F8775) for 10 min at 37 °C. After cross-linking, cells were lysed and sonicated to reduce the DNA length to 200-500 bp. Sheared chromatin was immunoprecipitated using 2 μg of the following Abs per sample: anti-LDB1 (Santa Cruz Biotechnology sc-365074) and its isotype control (R&D Systems MAB004), anti-GATA1 (Santa Cruz Biotechnology sc-265) and its isotype control (Santa Cruz Biotechnology, Inc. sc-3883), anti-TAL1 (Santa Cruz Biotechnology sc-393287), anti-LMO2 (Santa Cruz Biotechnology sc-65736), anti-E2A (Santa Cruz Biotechnology sc-416) and their isotype control (Thermo Fisher Scientific 14-4714-85). Immune complexes were then collected with protein A sepharose (Sigma-Aldrich GE17-5280-01) or protein G sepharose (Sigma-Aldrich GE17-0618-01) and extracted with an extraction buffer containing 1% SDS (Sigma-Aldrich L4509-500G) and 100 mM NaHCO_3_ (Sigma-Aldrich S5761-500G). The remaining procedures for the ChIP-qPCR assay, including DNA cross-link reversal, DNA extraction, and qPCR analysis, are detailed in an earlier publication [19]. The primer pairs used for ChIP-qPCR assays are detailed in Supplementary Table S1.

### 2.17 Luciferase assay

The enhancer regions covering a 210 bp DNA segment within the third intron (*Bcl11a* intron #3) or a 225 bp DNA segment within the fourth intron (*Bcl11a* intron #4) at the *Bcl11a* locus were cloned into a pGL4.23 vector (Promega E8411) using *Bam*HI and *Sal*I restriction sites to enhance the mRNA expression of Luc2 firefly luciferase (Fig. 6F). The cDNA encoding *Ldb1*, *Lmo2*, or *Gata1* was cloned into a pCDH-CMV-MCS-EF1-puro vector (System Biosciences CD510B-1) at the *Eco*RI and *Bam*HI, *Xba*I and *Eco*RI, or *Xba*I and *Bam*HI restriction sites, respectively. The pGL4.23 vector (100 ng) containing either *Bcl11a* intron #3 or *Bcl11a* intron #4, and the pRL-SV40 vector (Promega E2231, 100 ng) containing the internal *Renilla* luciferase were co-transfected, along with the indicated combinations of the pCDH-CMV-MCS-EF1-puro vector (100 ng) encoding *Ldb1*, *Lmo2*, or *Gata1*, into 1 × 10^4^ HEK293T cells (ATCC CRL-3216) in 96-well plates using Lipofectamine™ 2000 reagent (Fig. 6G). The empty pGL4.23 vector was also transfected as a baseline control for relative luciferase activity. Following transfection, HEK293T cells were cultured in DMEM (Welgene LM001-05) supplemented with 10% FBS and 1% penicillin/streptomycin. Forty-eight h post-transfection, each transfectant was lysed in passive lysis buffer (Promega E1910) for 30 min at room temperature. Firefly and *Renilla* luciferase activities were measured using the Dual-Glo® luciferase assay system (Promega E2920) according to the manufacturer's instructions. Relative luciferase activity was calculated as the ratio of firefly to *Renilla* luciferase activity. Each transfection was performed in quadruplicate and repeated three times, with the relative luciferase activity of the empty pGL4.23 vector set as the baseline value of “1”. The primers used for cloning the promoter constructs are detailed in Supplementary Table S1.

### 2.18 CRISPR/Cas9-mediated knock out of LDB1

A targeted guide RNA (gRNA) to knock out human *LDB1* was cloned into the lentiCRISPRv2GFP vector (Addgene 82416), which contains the Cas9 cDNA. K562 cells were transfected with either a mock (empty lentiCRISPRv2GFP vector) or knockout construct (gRNA-expressing lentiCRISPRv2GFP vector) using Lipofectamine™ 2000 reagent (Thermo Fisher Scientific 11668-019) according to the manufacturer's protocol. Two days after transfection, EGFP^high^ cells were sorted using a FACSAria™ II cell sorter (BD Biosciences), and individual clones were grown in 96-well plates in IMDM supplemented with 10% FBS and 1% penicillin/streptomycin. Genomic DNA from each clone was then isolated using the DNA purification kit (Cosmogenetech CMB-012) and sequenced to verify the knockout of human *LDB1* in individual K562 cell clones. The targeted gRNA sequence is shown in Supplementary Fig. S13A, and the sequencing results for the knockout cell line are shown in Supplementary Fig. S13B.

ROS levels, heme content, cell proliferation, and apoptosis in K562 cells transduced with either the mock or human *LDB1* knockout construct were analyzed using the same protocols described above.

### 2.19 Overexpression of Bcl11a in erythrocyte precursors

Full-length cDNAs of *Bcl11a* were cloned into the pCDH-CMV-MCS-EF1-CopGFP vector (System Biosciences CD511B-1) at the *Bam*HI and *Not*I restriction sites. The primers used for cloning the *Bcl11a* cDNA are detailed in Supplementary Table S1. Either empty vector or *Bcl11a*-expressing vector was co-transfected with packaging and envelope plasmid DNA into 293FT cells (Thermo Fisher Scientific R70007) using calcium phosphate transfection. Three days after the transfection, the culture supernatant containing viral particles was collected, filtered, and concentrated by centrifugation at 20,000*g* for 2 h at 4 °C. Subsequently, Ter119^-^ cells from E14.5 FL were transduced with either empty vector or *Bcl11a*-expressing vector in IMDM supplemented with 15% FBS, 1% penicillin/streptomycin, 2 mM _l_-glutamine, 100 μM 2-mercaptoethanol, 50 ng/ml recombinant mouse stem cell factor (Peprotech 250-03), 30 ng/ml recombinant human FLT-3L (R&D systems 308-FK-025), and 20 ng/ml recombinant mouse IL-6 (Sino biological 50136-MNAE) [27]. After 16 h, transduction efficiency was evaluated by analyzing GFP-positive cells using the FACSVerse™. The cells were then subjected to *in vitro* erythroid differentiation, cellular ROS analysis, cell cycle analysis, and apoptosis measurement as described above.

### 2.20 Statistics

A one-way ANOVA with Tukey HSD analysis was performed to compare mean values among three or more independent groups, and a two-tailed unpaired Student's *t*-test was used to compare mean values between two independent groups, using GraphPad Prism (GraphPad Software Inc). All data are presented as means ± SEM.

## 3 Results

### 3.1 LDB1 deficiency blocks erythropoiesis at the proerythroblast stage

To explore the intrinsic role of LDB1 during definitive erythropoiesis in the FL, we generated KO mice with a homozygous deletion of *Ldb1* in hematopoietic cells (Supplementary Fig. S1A and B). LDB1 was not detected by Western blot in the E14.5 KO FL (Supplementary Fig. S1C). *Ldb1* mRNA transcripts were also undetectable in various stages of HSPCs, including all stages of erythrocyte precursors (Supplementary Fig. S1D) in the E14.5 KO FL. Subsequently, we characterized phenotypes of KO mice compared to their littermate controls, WT mice.

No KO pups surviving beyond P5 were detected. P1 and P5 KO pups appeared smaller than their WT counterparts (Supplementary Fig. S2A). The thymi and spleens of P1 and P5 KO pups showed severe hypoplasia (Supplementary Fig. S2B). Notably, the spleens of P1 KO pups had a normal red color, whereas those of P5 KO pups were white and appeared to lack a visible red pulp zone, indicating that the cause of death was a gradual reduction in RBCs from P1 to P5 (Supplementary Fig. S2B). However, the E14.5 KO and WT fetuses were indistinguishable in appearance, and the size and absolute number of the E14.5 KO FLCs were comparable to those of WT fetuses (Supplementary Fig. S2A–C). Starting at E16.5, the KO FLCs exhibited lower cellularity than WT fetuses (Supplementary Fig. S2C). Accordingly, the absolute cell numbers in the thymi and spleens of KO mice were significantly lower at P1 and P3 than those of WT pups (Supplementary Fig. S2D and E). Therefore, hematopoietic cell-specific *Ldb1* disruption causes noticeable defects in hematopoiesis starting at E16.5, with significant manifestations by P1 and P3, which ultimately leading to the KO pups' failure to survive past P5 due to defects in definitive erythropoiesis.

Since defects in the hematopoietic lineage became evident by E16.5 KO FL, we analyzed the E14.5 WT and KO FLCs to establish a baseline and identify early-stage disruptions that could precede the more pronounced defects observed later. Unexpectedly, the percentages and absolute numbers of Lin^−^ cells in the FL of KO fetuses were significantly higher than those in the FL of WT fetuses at E14.5, while total FLC counts were comparable between WT and KO fetuses at the same developmental stage (Fig. 1A and B). Given these unexpected findings regarding Lin^−^ cell populations, we further investigated the composition of specific cell types within the E14.5 KO FL. Although the absolute number of LSK cells (Lin^−^Sca-1^+^c-kit^+^ cells) [20] was proportionally higher in the E14.5 KO FL, their percentage decreased compared to the WT FL (Fig. 1A and B). In contrast, both the frequency and absolute number of myeloid progenitors (Lin^−^Sca-1^−^c-kit^+^ cells) [20] were increased in the E14.5 KO FL compared to the WT FL (Fig. 1A and B). These results suggest that the increase in Lin^−^ cells in the E14.5 KO FL is primarily due to an expansion of myeloid progenitors rather than LSK cells.

To assess the role of LDB1 in the long-term reconstitution capacity of HSCs, we performed competitive repopulation assays using adoptive transfer of E14.5 FLCs from WT or KO CD45.2 fetuses, mixed with BMCs from WT CD45.1 mice, into lethally irradiated WT CD45.1 recipient mice (Supplementary Fig. S3A). At 12 weeks post-transplantation, KO FLCs showed impaired repopulation of HSPCs in the BM compared to WT FLCs (Supplementary Fig. S3B and C). These results confirm that LDB1 is essential for the functional integrity of LT-HSCs [13].

CMPs differentiate into GMPs or MEPs. The increase in the frequency and absolute numbers of myeloid progenitors in the E14.5 KO FL was accompanied by a marked rise in MEPs (Lin^−^Sca-1^−^c-kit^+^CD16/32^−^CD34^−^ cells), while CMPs (Lin^−^Sca-1^−^c-kit^+^CD16/32^−^CD34^+^ cells) and GMPs (Lin^−^Sca-1^−^c-kit^+^CD16/32^+^CD34^+^ cells) were significantly reduced in E14.5 KO FL compared to those in WT FL (Fig. 1C and D). To validate these findings, we conducted a CFU assay using sorted CMPs from the E14.5 WT and KO FLCs and found that the absolute numbers of CFU-granulocyte, erythrocyte, macrophage, megakaryocyte (CFU-GEMM), and CFU-granulocyte and/or macrophage (CFU-GM) were significantly decreased while the numbers of burst-forming unit-erythroid (BFU-E), an early erythroid progenitor, remained comparable to WT levels (Fig. 1E). These results imply that most of the accumulated MEPs in the E14.5 KO FL are erythroid progenitors that are unable to differentiate into erythrocytes. Additionally, both the frequency and absolute numbers of megakaryocyte precursors in the E14.5 KO FL were reduced compared to those in the WT FL (Supplementary Fig. S4).

To further delineate the developmental stage of erythrocytes affected in the E14.5 KO FL, we analyzed erythrocyte precursors according to their expression of Ter119 and CD71 [26,33,34]. Consistent with the BFU-E results, the frequency and absolute numbers of early erythroid progenitors (Ter119^−^CD71^low/med^ cells, S0 cells) from the E14.5 KO FL were comparable to those of the WT FL (Fig. 1F and G). Interestingly, the frequency and absolute numbers of proerythroblasts (Ter119^−^CD71^high^ cells, S1 cells) in the E14.5 KO FL were significantly increased compared to those in the WT FL (Fig. 1F and G). Conversely, the frequency and absolute number of basophilic erythroblasts (Ter119^+^CD71^high^ cells, S2 cells), late basophilic and polychromatophilic erythroblasts (Ter119^+^CD71^med^ cells, S3 cells), and orthochromatophilic erythroblasts (Ter119^+^CD71^low^ cells, S4 cells) were markedly decreased in the E14.5 KO FL compared to those in the WT FL (Fig. 1F and G). Collectively, these results indicate that hematopoietic cell-specific *Ldb1* deletion causes an accumulation of proerythroblasts, impairing their progression to fully mature erythrocytes.

### 3.2 LDB1 deficiency in proerythroblasts causes apoptosis due to ROS accumulation from excessive heme content

**Fig. 2:**
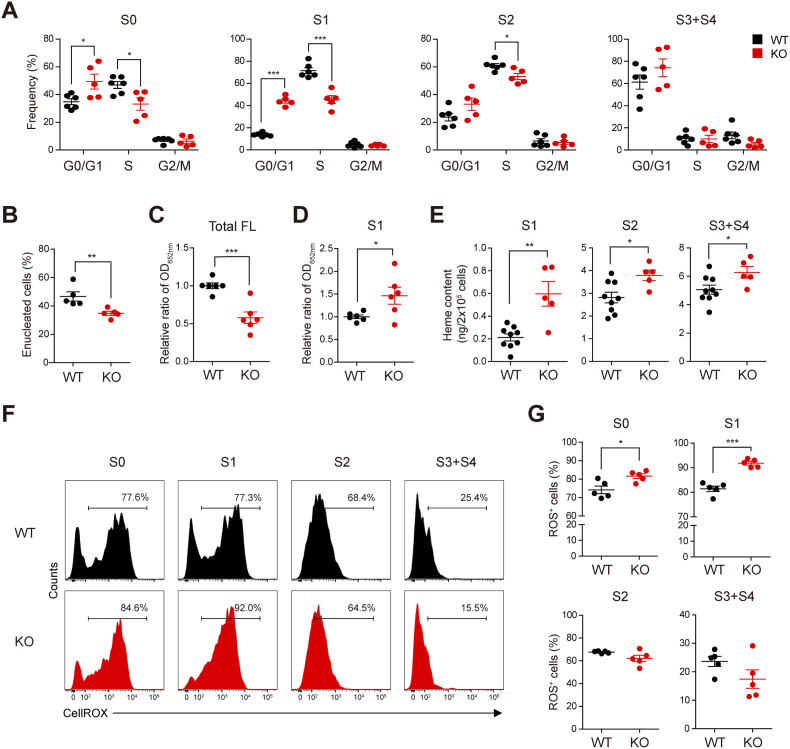
LDB1 deficiency in erythrocyte precursors causes cell cycle arrest and apoptosis due to the accumulation of ROS from excess heme content. (**A**) Frequency of different cell cycle phases in erythrocyte precursors at each stage (S0 to S4 cells) from E14.5 WT (*n* = 6) and KO (*n* = 5) FLs. (**B**) Percentage of enucleated cells from E14.5 WT and KO FLs was evaluated after Giemsa staining. *n* = 5. (**C** and **D**) Relative intensity of benzidine staining of total FLCs (Total FL) (**C**), and S1 cells (S1) (**D**) from E14.5 WT and KO fetuses. *n* = 6. (**E**) Cellular heme content in erythrocyte precursors at each stage from E14.5 WT (*n* = 9) and KO (*n* = 5) FLs were quantified using an oxalic acid assay. (**F** and **G**) Representative flow cytometry plots (**F**) and frequency of ROS^+^ cells (**G**) in erythrocyte precursors at each stage from E14.5 WT and KO FLs. *n* = 5. Statistical significance was assessed by two-tailed Student's *t-*test. ∗*P* < 0.05; ∗∗*P* < 0.01; ∗∗∗*P* < 0.001. All data are presented as the mean ± SEM.

During erythrocyte maturation, active cell cycle progression occurs in proerythroblasts (S1 cells). About 50% of S0 cells are in the S-phase, whereas most S1 cells are in the S-phase [34]. This is further supported by the significantly higher expression of E cyclins and the marked downregulation of p57 in S1 cells compared to S0 cells [34]. After the S1 stage, the proportion of cells in the S-phase gradually decreases in both S2 and S3 cells [34]. In the E14.5 FL, S0 and S1 cells from the KO showed a significant reduction in S-phase progression with a marked increase in G0/G1-phase compared to WT cells (Fig. 2A, and Supplementary Fig. S5). These results indicate that erythrocyte maturation in the E14.5 KO FL might be impaired, potentially in association with a block in the cell cycle transition from the G0/G1-phase to the S-phase in proerythroblasts.

To test this hypothesis, we measured the percentage of enucleated cells and performed benzidine staining on the E14.5 WT and KO FLCs. The results showed a lower percentage of total enucleated cells (Fig. 2B, and Supplementary Fig. S6) and reduced benzidine staining intensity, with fewer benzidine-positive cells in the KO FL compared to the WT FL (Fig. 2C, and Supplementary Fig. S7A). Unexpectedly, S1 cells in the E14.5 KO FL exhibited higher benzidine staining intensity, indicating greater heme accumulation compared to those in the WT FL (Fig. 2D, and Supplementary Fig. S7B). To quantify cellular heme concentrations, oxalic acid staining was performed on immature erythrocytes at each developmental stage in the E14.5 WT and KO FLs. Consistent with the benzidine staining results, cellular heme content was significantly higher in KO precursor cells compared to WT cells (Fig. 2E). Precise regulation of heme synthesis and degradation is essential for erythroid maturation and cell survival, as excessive heme accumulation in proerythroblasts induces oxidative stress, leading to apoptosis [35]. We found that intracellular ROS levels were significantly increased in both S0 and S1 cells of the E14.5 KO FL compared to those in the WT FL (Fig. 2F and G). To determine whether excessive heme accumulation in KO proerythroblasts results from increased synthesis or impaired degradation, we compared relative heme synthesis and degradation between WT and KO proerythroblasts. The results clearly showed that impaired heme degradation in KO proerythroblasts leads to heme accumulation (Supplementary Fig. S8). Overall, these results suggest that LDB1 may contribute to heme regulation, and its deficiency is associated with oxidative stress in proerythroblasts from E14.5 FLs.

**Fig. 3:**
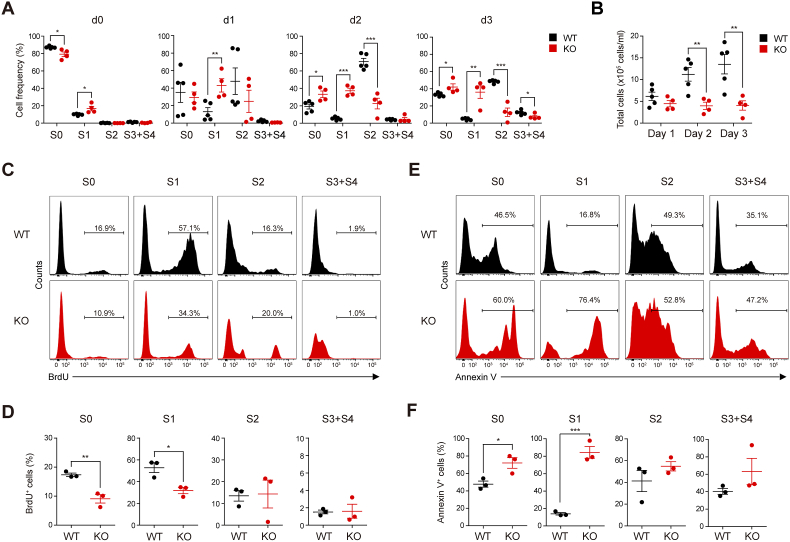
LDB1 deficiency impairs *in vitro* erythroid differentiation by reducing cell proliferation and increasing apoptosis. Ter119^-^ cells (S0 and S1 cells) from E14.5 WT and KO FLCs were cultured for 3 days to induce erythroid differentiation. (**A** and **B**) Frequency of erythrocyte precursors at each stage (S0 to S4 cells) (**A**) and absolute number of total erythrocyte precursors (**B**) at the indicated time points during erythroid differentiation (WT, *n* = 5; KO, *n* = 4). d, days after culture. (**C** and **D**) Representative flow cytometry plots (**C**) and frequency of BrdU^+^ cells (**D**) at each stage of erythrocyte precursors on day 3 after culture. *n* = 3. (**E** and **F**) Representative flow cytometry plots (**E**) and frequency of Annexin V^+^ cells (**F**) at each stage of erythrocyte precursors on day 3 of culture. *n* = 3. Statistical significance was assessed by two-tailed Student's *t-*test. ∗*P* < 0.05; ∗∗*P* < 0.01; ∗∗∗*P* < 0.001. All data are presented as the mean ± SEM.

Next, we conducted *in vitro* erythroid differentiation assays to visualize the step-by-step process of erythroid differentiation using Ter119^-^ cells (S0 and S1 cells) from E14.5 FLs [26]. At the beginning of the culture (d 0), the proportion of S0 to S1 cells in WT Ter119^-^ cells was approximately 9:1, whereas it was nearly 5:1 in KO Ter119^-^ cells (Fig. 3A, and Supplementary Fig. S9). The proportion of WT S2 cells increased after culture, more than doubling relative to WT S1 cells on days 2 and 3 (Fig. 3A, and Supplementary Fig. S9). These results indicate that WT proerythroblasts differentiated properly into late erythroblasts. In contrast, the proportion of KO S2 cells never exceeded that of KO S1 cells throughout the culture period, with KO S1 cells remaining the predominant population on days 1 and 2 (Fig. 3A, and Supplementary Fig. S9). These findings confirm that KO proerythroblasts fail to differentiate into mature erythrocytes due to a block in the transition from S1 to S2 stages. Consistent with these observations, the total number of KO erythroblasts did not increase throughout the culture period, whereas the number of WT erythroblasts gradually increased (Fig. 3B). Consequently, the proliferation of most KO erythrocyte precursors was reduced compared to that of WT cells on day 3 of culture (Fig. 3C and D), and the percentage of apoptotic cells was significantly higher in KO S0 and S1 cells than in WT cells at this time point (Fig. 3E and F). To test whether treatment with the ROS scavenger (NAC) enhances the survival and proliferation of KO erythroblasts, Ter119^-^ cells (S0 and S1 cells) from E14.5 WT and KO FLs were cultured for 3 days in the presence of 1 mM or 2.5 mM NAC. Flow cytometric analysis showed an increased frequency of mature erythroid precursors (S3 and S4 cells) and a corresponding decrease in immature populations (S0 and S1 cells) after NAC treatment compared with untreated KO cells (Supplementary Fig. S10A and B). BrdU incorporation assays demonstrated enhanced proliferation across erythroid stages, whereas Annexin V staining revealed a significant reduction in apoptosis in NAC-treated KO cells (Supplementary Fig. S10C and D). These rescue effects confirm that elevated ROS contributes directly to the erythroid defects caused by LDB1 loss and support the causal relationship between oxidative stress and impaired differentiation. Overall, these results suggest that *Ldb1* deficiency impairs erythrocyte maturation in the E14.5 FL, accompanied by disrupted cell cycle progression in proerythroblasts and excessive heme accumulation.

### 3.3 LDB1 transcriptionally activates fetal β-globin repressor genes such as Bcl11a, Cbfa2t3, and Sox6

**Fig. 4:**
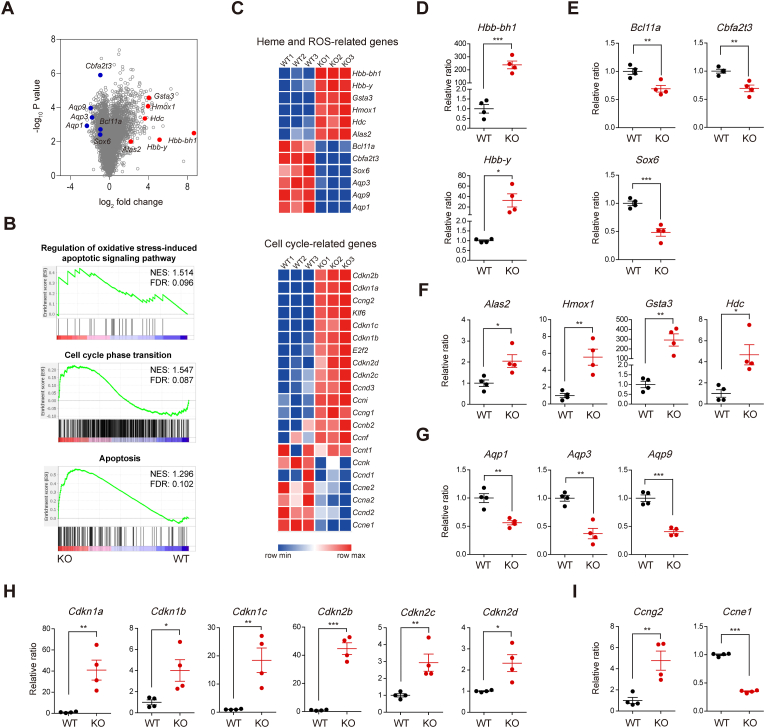
Differentially expressed gene (DEG) profiles between E14.5 WT and KO FL-derived proerythroblasts. (**A**-**C**) RNA-seq analyses were performed using total RNA from proerythroblasts (S1 cells) of either E14.5 WT or KO FLs. Volcano plot presenting DEGs between WT and KO S1 cells (**A**), gene set enrichment analysis (GSEA) profiles of DEGs between WT and KO S1 cells (**B**), and heatmap plots showing the expression patterns of heme and ROS-related genes or cell cycle-related genes between WT and KO S1 cells (**C**). (**D**-**I**) mRNA expression patterns of mouse embryonic β-globin genes (**D**), fetal globin repressor genes (**E**), genes that regulate heme biosynthesis and oxidative stress responses (**F**), aquaporin genes involved in regulating ROS transport (**G**), tumor suppressor genes (**H**), and cyclins (**I**) in S1 cells derived from either E14.5 WT or KO FLCs were assessed by qRT-PCR. *n* = 4. Statistical significance was assessed by two-tailed Student's *t-*test. ∗*P* < 0.05; ∗∗*P* < 0.01; ∗∗∗*P* < 0.001. All data are presented as the mean ± SEM.

To explore the mechanism by which LDB1 plays a role in erythrocyte maturation, we conducted RNA-seq to compare gene expression patterns between WT and KO S1 cells from the E14.5 FL. This analysis revealed that 1302 downregulated genes and 1017 upregulated genes (fold change ≥1.5, *p* < 0.05) in KO S1 cells compared to their WT counterparts [Gene Expression Omnibus (GEO) accession: GSE247801] (Fig. 4A). Gene set enrichment analysis (GSEA) further revealed that genes involved in oxidative stress responses, cell cycle phase transition, and apoptosis were significantly altered in KO S1 cells compared to WT S1 cells (Fig. 4B).

A heat map based on RNA-seq analyses confirmed these findings, showing increased expression of embryonic β-globin genes (*Hbb-bh1* and *Hbb-y*) [4,5], the heme synthesis gene (*Alas2*) [36], and ROS-inducible antioxidant genes (*Gsta3*, *Hdc* and *Hmox1*) [25,37,38] in KO S1 cells. Conversely, genes associated with fetal globin repression (*Bcl11a, Cbfa2t3*, and *Sox6*) [[39], [40], [41]] and ROS transport (*Aqp1, Aqp3*, and *Aqp9*) [42] were decreased in KO S1 cells compared to WT S1 cells (Fig. 4C). However, the expression of adult β-globin genes (*Hbb-b1* and *Hbb-b2*) [4,5] was not statistically different between WT and KO S1 cells (GEO accession: GSE247801). The heat map also revealed that genes involved in cell cycle progression [43] were downregulated, while tumor suppressor genes [44] were upregulated in KO S1 cells (Fig. 4C).

To validate the RNA-seq results, we performed qRT-PCR using total RNA from both WT and KO S1 cells. Notably, *Hbb-bh1* expression was more than 200-fold higher, and *Hbb-y* expression was more than 30-fold higher in KO S1 cells compared to WT S1 cells (Fig. 4D). Flow cytometry analysis confirmed that embryonic ε-globin (HBBY) levels in KO S1 cells were significantly higher than those in WT S1 cells (Supplementary Fig. S11). Consistent with these findings, fetal globin repressor genes such as *Bcl11a, Cbfa2t3,* and *Sox6* were significantly downregulated in KO S1 cells compared to WT S1 cells (Fig. 4E). These results might suggest that KO S1 cells significantly overexpress embryonic β-globin genes due to the downregulation of fetal globin repressor genes. Consistent with RNA-seq data, qRT-PCR results revealed elevated mRNA expression levels of *Alas2*, *Hmox1*, *Gsta3*, and *Hdc* genes in KO S1 cells compared to WT S1 cells (Fig. 4F). These genes are known to be involved in heme biosynthesis and oxidative stress responses [25,[36], [37], [38]]. Moreover, expression levels of aquaporin genes such as *Aqp1*, *Aqp3*, and *Aqp9* involved in regulating ROS transport were reduced in KO S1 cells compared to WT S1 cells [42] (Fig. 4G). Additionally, the expression levels of several tumor suppressor genes, including *Cdkn1a* (p21), *Cdkn1b* (p27), *Cdkn1c* (p57), *Cdkn2b* (p15), *Cdkn2c* (p18), and *Cdkn2d* (p19), were significantly upregulated in KO S1 cells compared to WT S1 cells (Fig. 4H). In parallel, *Ccng2* (cyclin G2), a cell cycle inhibitory cyclin [45], was upregulated, whereas *Ccne1* (cyclin E1), a facilitator for the G1 to S-phase transition [46], was downregulated in KO S1 cells relative to WT S1 cells (Fig. 4I). Taken together, these results indicate that *Ldb1* deficiency in S1 cells could lead to the downregulation of fetal globin repressor genes, resulting in excessive production of embryonic globins and the subsequent accumulation of hemoglobin. The elevated hemoglobin levels in KO S1 cells might contribute to cell cycle arrest and apoptosis through oxidative stress.

**Fig. 5:**
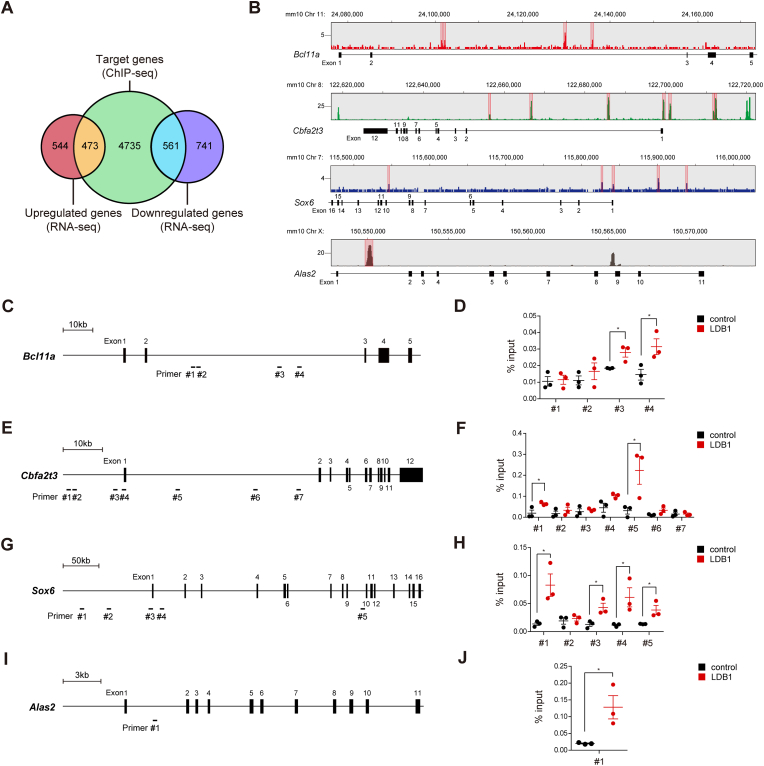
LDB1 binds to the *Bcl11a*, *Cbfa2t3* and *Sox6* gene loci. (**A**) Venn diagram illustrating the number of putative LDB1 target genes based on the overlap between genes bound by LDB1 within the ±20 kb of the transcription start site (TSS) and upregulated or downregulated genes in E14.5 KO proerythroblasts (S1 cells) compared to WT. (**B**) Integrative genomics viewer tracking the putative binding sites of LDB1 at the *Bcl11a*, *Cbfa2t3*, *Sox6*, and *Alas2* loci. (**C**-**J**) ChIP-qPCR assays were performed to assess LDB1 occupancy at respective gene loci in E14.5 FLCs. The locations of each indicated primer set within the *Bcl11a*, *Cbfa2t3*, *Sox6*, and *Alas2* loci are shown in the schematic diagrams (**C**, **E**, **G** and **I**) along with the corresponding ChIP-qPCR results (**D**, **F**, **H** and **J**). *n* = 3. Control, isotype control Ab; LDB1, anti-LDB1 Ab. Statistical significance was assessed by two-tailed Student's *t*-test. ∗*P* < 0.05. All data are presented as the mean ± SEM.

To identify specific LDB1 target loci involved in erythrocyte maturation, we analyzed a ChIP-seq dataset from MEL cells using an anti-LDB1 Ab [9] and identified 5769 genes with LDB1 occupancy near their enhancer regions (±20 kb from the transcription start site, TSS). By integrating ChIP-seq with RNA-seq data, we identified 473 upregulated genes and 561 downregulated genes among the putative genes directly regulated by LDB1 (Fig. 5A). Intriguingly, *Bcl11a*, *Cbfa2t3*, and *Sox6*, which were downregulated in KO S1 cells, were identified as potential LDB1 target genes (Fig. 5B). The *Alas2* locus, previously reported as a direct LDB1 binding target [36], also exhibited LDB1 binding peaks (Fig. 5B). To determine the specific DNA regions bound by LDB1 in the *Bcl11a, Cbfa2t3, Sox6,* and *Alas2* loci, we performed a ChIP-qPCR assay using primer pairs targeting the putative LDB1 binding sites within each gene locus. This analysis revealed strong LDB1 binding at the second intron region of the *Bcl11a* locus (Fig. 5C and D, primers #3 and #4), the 5′ distal region of the TSS and the first intron regions of the *Cbfa2t3* locus (Fig. 5E and F, primers #1 and #5), the 5′ distal region of the TSS, the first exon, the first intron, and intron 9 regions of the *Sox6* locus (Fig. 5G and H, primers #1, #3, #4, and #5), and the first intron region of the *Alas2* locus (Fig. 5I and J, primer #1).

### 3.4 The LDB1 complex, including LMO2 and GATA1, binds BCL11A enhancers to promote transcription in humans and mice

**Fig. 6:**
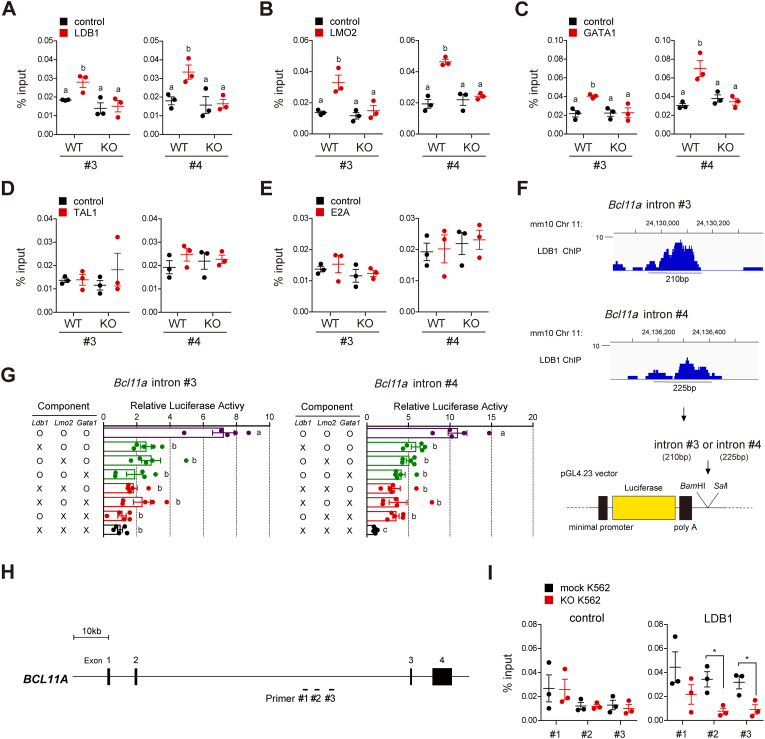
The LDB1 complex containing LMO2 and GATA1 directly enhances the transcription of the *Bcl11a* gene. (**A-E**) ChIP-qPCR assays were performed to assess the occupancy of putative LDB1 complex component proteins, including LDB1, LMO2, GATA1, TAL1, and E2A, at the *Bcl11a* locus in E14.5 WT and KO FLCs. *n* = 3. Control, isotype control Ab; LDB1, anti-LDB1 Ab; LMO2, anti-LMO2 Ab; GATA1, anti-GATA1 Ab; TAL1, anti-TAL1 Ab, E2A, anti-E2A Ab; WT, E14.5 FLCs; KO, E14.5 KO FLCs. Statistical significance was assessed by one-way ANOVA with Tukey HSD analysis. Mean values not sharing the same superscript letter (^a, b^) differ significantly at *P* < 0.05. (**F** and **G**) Dual-luciferase reporter assays were performed using the 210 bp region covering primer #3 (*Bcl11a* intron #3) or the 225 bp region covering primer #4 (*Bcl11a* intron #4) at *Bcl11a* locus. The relative luciferase activity of each construct containing either *Bcl11a* intron #3 or *Bcl11a* intron #4 was measured in HEK293T cells transfected with the indicated combination of each expression vector encoding *Ldb1*, *Lmo2*, or *Gata1* cDNA. The schematic diagrams of each cloned enhancer region (**F**) and the results of the luciferase reporter assays (**G**) are presented. *Ldb1*, expression vector encoding *Ldb1* cDNA; *Lmo2*, expression vector encoding *Lmo2* cDNA; *Gata1*, expression vector encoding *Gata1* cDNA; ◯, transfected cells; ☓, untransfected cells. *n* = 5. Statistical significance was assessed by one-way ANOVA with Tukey HSD analysis. Mean values not sharing the same superscript letter (^a, b, c^) differ significantly at *P* < 0.05. (**H** and **I**) ChIP-qPCR assays were performed to assess LDB1 occupancy at the human *BCL11A* locus in K562 cells. The locations of each indicated primer set within the *BCL11A* locus are shown in the schematic diagram (**H**) along with the corresponding ChIP-qPCR results (**I**). *n* = 3. Mock K562, empty vector transfected K562 cells; KO K562, human *LDB1* knockout K562 cells; Control, isotype control Ab; LDB1, anti-LDB1 Ab. Statistical significance was assessed by two-tailed Student's *t*-test. ∗*P* < 0.05. All data are presented as the mean ± SEM.

Since our findings indicated that LDB1 could directly increase *Bcl11a* mRNA expression, we investigated whether LDB1 deficiency could reduce BCL11A expression in KO S1 cells compared to WT S1 cells. Flow cytometry analysis confirmed that BCL11A levels in KO S1 cells were significantly lower than those in WT S1 cells (Supplementary Fig. S12). To characterize the core LDB1 complex regulating the transcriptional activity of *Bcl11a*, we performed additional ChIP-qPCR assays targeting the binding sites of primers #3 and #4, using Abs against LMO2, GATA1, TAL1, and E2A. Direct binding by LDB1 complex members, including LDB1, LMO2, and GATA1, was observed in the region covered by primers #3 and #4 (Fig. 6A–C). However, no binding signals were detected for TAL1 or E2A (Fig. 6D and E). Next, we performed dual-luciferase reporter assays using the 210 bp region covering primer #3 or the 225 bp region covering primer #4 to provide direct evidence that the LDB1 complex could enhance the transcription of *Bcl11a* mRNA by binding to its enhancer regions (Fig. 6F and G). Each luciferase reporter construct, containing a specific enhancer region, was cloned after the poly A tail of the minimal promoter-driven luciferase gene using *Bam*HI and *Sal*I restriction sites, and then transfected into HEK293T cells (Fig. 6F). Subsequently, different combinations of LDB1, LMO2, and GATA1 were expressed in the HEK293T cells harboring the luciferase reporter constructs (Fig. 6G). The relative luciferase activity in these cells was then measured. In HEK293T cells, all three proteins were required to efficiently promote the transcriptional activity of each enhancer region (Fig. 6G). The combined results clearly demonstrate that the LDB1 complex, containing LMO2 and GATA1, directly promotes the transcription of *Bcl11a* by binding to its enhancer regions.

To confirm the relevance of these findings in humans, we used CRISPR/Cas9 to knock out *LDB1* in human erythroleukemia cells (K562 cells) and measured BCL11A expression levels by Western blot, comparing empty vector transfectants (mock K562) with *LDB1* knockout cells (KO K562). The results clearly showed that LDB1 enhances the mRNA expression of *BCL11A* in K562 cells (Supplementary Fig. S13A–C). Previous studies have demonstrated that human BCL11A directly enhances mRNA transcription of the human fetal globin gene (*HBG*) [47]. Consistent with these results, the mRNA transcript of the human fetal globin gene (*HBG*) was upregulated in KO K562 compared to that in mock K562 (Supplementary Fig. S13D). Flow cytometry analysis further confirmed increased protein levels of human embryonic ε-globin (HBE1) and fetal hemoglobin (HbF) in KO K562 cells (Supplementary Fig. S13E–H). Consistent with the phenotype observed in erythroblasts from E14.5 KO FLs, KO K562 cells also showed reduced proliferation and increased apoptosis associated with elevated levels of ROS and heme content compared with mock K562 cells (Supplementary Fig. S13I–L). Next, we performed ChIP-qPCR assays in K562 cells using primer pairs targeting the putative LDB1 binding sites within the human *BCL11A* locus. To design specific primer pairs, we analyzed histone marks from previous ChIP-seq data in primary human erythroblasts (GEO accession: GSE36994) [48] and identified three putative enhancer regions within intron 2 of the human *BCL11A* locus (Supplementary Fig. S14). Subsequent ChIP-qPCR assays in K562 cells revealed that LDB1 could bind to the enhancer regions covered by primers #2 and #3 (Fig. 6H and I). These results support a conserved role of LDB1 in the transcriptional activation of *BCL11A* in humans.

### 3.5 BCL11A promotes differentiation of Ldb1^−/−^ proerythroblasts by rescuing them from ROS-mediated apoptosis

**Fig. 7:**
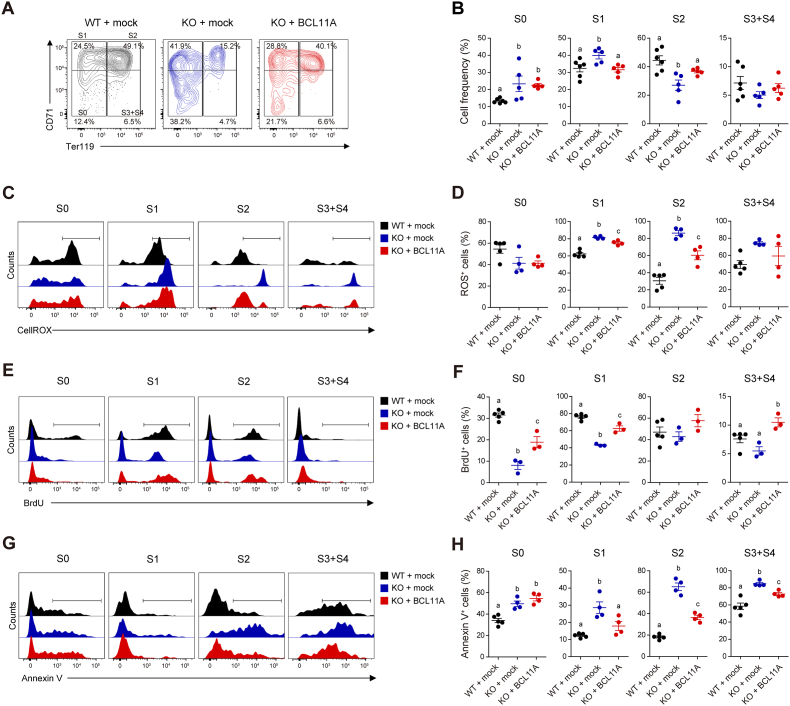
Ectopic expression of BCL11A facilitates the differentiation of KO erythrocyte precursors. Ter119^-^ cells (S0 and S1 cells) from E14.5 WT and KO FLs were transduced with either an empty vector (mock) or an expression vector encoding *Bcl11a* cDNA (BCL11A), followed by *in vitro* erythroid differentiation assays for 3 days. (**A** and **B**) Representative flow cytometry plots (**A**) and frequency of erythrocyte precursors at each stage (**B**) on day 3 of culture (WT + mock, *n* = 6; KO + mock, *n* = 5; KO + BCL11A, *n* = 5). (**C** and **D**) Representative flow cytometry plots (**C**) and frequency of ROS^+^ cells (**D**) at each stage of erythrocyte precursors on day 3 of culture (WT + mock, *n* = 5; KO + mock, *n* = 4; KO + BCL11A, *n* = 4). (**E** and **F**) Representative flow cytometry plots (**E**) and frequency of BrdU^+^ cells (**F**) at each stage of erythrocyte precursors on day 3 of culture (WT + mock, *n* = 5; KO + mock, *n* = 3; KO + BCL11A, *n* = 3). (**G** and **H**) Representative flow cytometry plots (**G**) and frequency of Annexin V^+^ cells (**H**) at each stage of erythrocyte precursors on day 3 of culture (WT + mock, *n* = 5; KO + mock, *n* = 4; KO + BCL11A, *n* = 4). Statistical significance was assessed by one-way ANOVA with Tukey HSD analysis. Mean values not sharing the same superscript letter (^a, b, c^) differ significantly at *P* < 0.05. All data are presented as the mean ± SEM.

To test whether ectopic expression of *Bcl11a* could rescue erythroid maturation in Ter119^-^ cells (S0 and S1 cells) from E14.5 KO FLs, we conducted lentiviral *Bcl11a* gene transfer in these cells (Supplementary Fig. S15) and performed *in vitro* erythroid differentiation assays to compare the phenotypic changes in KO Ter119^-^ cells with those in WT Ter119^-^ cells. Three days after culture, the enforced expression of *Bcl11a* partially rescued erythroid maturation in KO proerythroblasts (S1 cells) compared to WT S1 cells (Fig. 7A and B). ROS levels in KO S1 cells were also substantially decreased by exogenous *Bcl11a* expression (Fig. 7C and D). We then examined cell proliferation and apoptosis during *in vitro* erythroid differentiation. Cell proliferation in KO S1 cells was partially restored by *Bcl11a* overexpression (Fig. 7E and F). Correspondingly, a reduction in apoptotic cells was observed in *Bcl11a*-transduced KO S1 and S2 cells (Fig. 7G and H). Taken together, these results suggest that ectopic expression of *Bcl11a* in KO proerythroblasts could facilitate erythroid differentiation, potentially by alleviating ROS-mediated apoptosis and supporting cell cycle progression.

## 4 Discussion

The regulation of hemoglobin switching is orchestrated by a complex network of transcription factors and co-regulators that modulate gene expression [4,5]. Central to this network is LDB1 which forms a multiprotein complex with other transcription factors, including LMO2 and GATA1. LDB1 plays a crucial role in regulating globin gene expression during erythropoiesis [[8], [9], [10], [11], [12]]. Previous studies have shown that the LDB1 complex is essential for the transcriptional activation of adult β-globin, and that forced chromatin looping via the LDB1 complex facilitates fetal β-globin expression, highlighting its pivotal role in globin gene regulation [49,50]. However, the precise mechanisms by which LDB1 regulates the transition from fetal to adult hemoglobin are not fully elucidated.

In this study, we present comprehensive findings that delineate the role of LDB1 in modulating the expression of fetal globin genes by characterizing mice lacking *Ldb1* selectively in erythrocyte precursors, combined with transcriptional, epigenomic, and promoter binding analyses. Our investigation reveals that the LDB1 complex, encompassing LMO2 and GATA1, is a direct transcriptional activator of fetal β-globin repressor genes including *Bcl11a*, *Cbfa2t3*, and *Sox6* in proerythroblasts. Loss of LDB1 in these cells is associated with increased heme content and elevated ROS levels, triggering cell cycle arrest and apoptosis, as indicated by the upregulation of heme/oxidative stress-related genes and tumor suppressor genes. Consistent with these observations, BCL11A expression in *Ldb1*-deficient proerythroblasts enhances erythroid differentiation by suppressing ROS-mediated apoptosis, thereby facilitating cell cycle progression. These findings highlight an important role for LDB1 during erythrocyte differentiation, as it may contribute to the proper balance of β-globin gene expression through transcriptional activation of *BCL11A*, thereby preventing globin chain imbalance and the associated oxidative stress, rather than acting through direct regulation of heme synthesis or degradation genes.

The phenotype of the KO mice displays several similarities to those observed in mice with a conditional deletion of *Ldb1* using Tie2cre and Mx1cre but also exhibits key discrepancies in fetal definitive erythropoiesis. Like these models, the KO mice exhibited defects in LT-HSCs, megakaryocytes, and erythrocytes [[11], [12], [13]]. In contrast to previous models, the total cellularity of E14.5 KO FLs was comparable to that of WT FLs, while MEPs in E14.5 KO FLs were markedly increased compared to those in WT FLs. This discrepancy led to the identification of novel LDB1 targets, specifically fetal globin repressor genes. Our combined RNA-seq and ChIP-seq analyses uncovered that the fetal globin repressor genes, including *Bcl11a*, *Cbfa2t3* (*Eto2*), and *Sox6*, are direct targets of LDB1.

BCL11A, a zinc finger transcriptional repressor, inhibits fetal β-globin gene expression by occupying essential regulatory sites within the β-globin gene cluster [39,47,51,52]. Indeed, our genomic sequence analysis using the SnapGene tool (https://www.snapgene.com/) revealed that the putative BCL11A binding sites (TGACCA motif) [47,52] are well conserved in mouse embryonic β-globin genes (*Hbb-bh1* and *Hbb-y*), as well as in human embryonic β-globin gene (*HBE1*) and fetal globin genes (*HBG1* and *HBG2*) (Supplementary Fig. S16). Moreover, BCL11A physically interacts with SOX6 [39], reinforcing its role in fetal globin repression. SOX6 has been shown to bind to the promoter of the *Hbb-y* gene, leading to its silencing in definitive erythroid cells [53]. Given their interaction, BCL11A and SOX6 likely cooperate at multiple regulatory sites within the β-globin locus to ensure robust repression of fetal globin genes [39,54]. In addition to BCL11A and SOX6, ETO2, the gene product of *Cbfa2t3*, contributes to the repression of fetal globin genes by either antagonizing the activity of the LDB1 complex or interacting with the NCOR1/SMRT co-repressor complex [55].

Based on our data, the expression levels of other fetal globin transcriptional repressor genes, including *Zbtb7a* [56], *Dnmt1* [57], *Eif2ak1* [58], and *Pogz* [59], were not significantly different between WT and KO S1 cells (GEO accession: GSE247801). Similarly, the expression levels of *Epo* and *Epor*, key components of a signaling pathway essential for the S-phase transition in S1-stage cells [34], were not significantly altered in KO S1 cells compared to those in WT cells (GEO accession: GSE247801).

Supporting our observation, the phenotype of the KO mice closely resembles that of *Bcl11a*, *Cbfa2t3* (*Eto2*), and *Sox6* deficient mice [40,53,[60], [61], [62]]. Among these, the phenotype of *Bcl11a* deficient mice most closely matches that of the KO mice [60,61]. These mice die during the perinatal period [60] and exhibit a 70- and 350-fold upregulation of the *Hbb-y* and *Hbb-bh1* genes, respectively, in E14.5 FLs compared to WT FLs [61]. *Cbfa2t3* deficient mice exhibit much milder erythrocyte defects than the KO mice. They are viable, fertile, and display only mild anemia at 8 weeks of age [40]. Interestingly, the phenotype of *Cbfa2t3* deficient FLs at E14.5 is also highly similar to that of the KO mice. At E14.5, the FLs of *Cbfa2t3* deficient fetuses display no difference in cellularity relative to wild-type mice, although they show a two-fold increase in S0 and S1 cells with a substantial decrease in S2 and S3 cells, along with markedly increased *Hbb-y* mRNA expression [40]. The proerythroblasts from E14.5 *Sox6* deficient FLs also mirror the phenotype of the KO proerythroblasts [53,62]. They display an accumulation of proerythroblasts (S1 cells) that fail to mature into S2 and S3 cell populations due to impeded cell cycle transitions, accompanied by markedly increased *Hbb-y* and *Hbb-bh1* mRNA expression [53,62].

The therapeutic and diagnostic potential of our findings on human disorders with ineffective erythropoiesis was further evaluated. First, our data highlight several putative gene-editing target sequences for gene therapy in β-thalassemia. Currently, targeted inactivation of human *BCL11A* is a promising strategy to increase fetal hemoglobin (HbF) production in β-thalassemia patients [[63], [64], [65], [66]]. Indeed, recent clinical studies have demonstrated that CRISPR/Cas9-mediated deletion of the erythroid enhancer region of *BCL11A* effectively restores HbF expression in β-thalassemia patients [65,66]. The enhancer region in human *BCL11A* regulated by LDB1 identified in this study (region covered by primer #2 in Fig. 6H) corresponds to the *BCL11A* erythroid enhancer region targeted by CRISPR/Cas9 [63,65]. The sequence of the mouse *Bcl11a* erythroid enhancer region is consistent with our findings (region covered by primers #3 and #4 in Fig. 5C and D, and Fig. 6A–G) [65,66]. Moreover, our study suggests two additional gene-editing target sequences for β-thalassemia patients by revealing enhancer regions regulated by LDB1 in the *Cbfa2t3* (region covered by primers #1 and #5 in Fig. 5E and F) and *Sox6* (region covered by primers #1, #3, #4, and #5 in Fig. 5G and H) loci. Combining our results with recent clinical findings, we suggest that the targeted deletion of all three enhancer regions regulated by LDB1 in the *BCL11A*, *CBFA2T3* and *SOX6* loci could be explored for β-thalassemia therapy, compared to the current focus on a single *BCL11A* enhancer region. Second, the KO mice may serve as a model system for investigating therapeutic interventions and identifying novel diagnostic biomarkers for human diseases associated with ineffective erythropoiesis, as their phenotypic characteristics align with several congenital erythrocyte disorders in humans. In β-thalassemia, the accumulation of unpaired α-globin chains increases heme levels and generates excessive ROS, causing oxidative damage and erythrocyte apoptosis [6,7]. Similarly, in KO erythroblasts, unpaired embryonic β-globins mimic the effects of α-globin chains in β-thalassemia, contributing to oxidative stress and cell death. The disease mechanism in KO mice share similarities with those observed in sideroblastic anemia, where excess heme intermediates cause ROS-mediated apoptosis of proerythroblasts [67].

Several limitations of this study should be considered. Although rescue experiments using NAC support a causal role for ROS in the observed phenotypes, the precise sequence linking embryonic globin overexpression, heme accumulation, ROS generation, and apoptosis remains inferential. This relationship should therefore be interpreted with caution. The use of HEK293T cells for enhancer–reporter assays represents a technical compromise chosen for their high transfection efficiency, and the results therefore indicate enhancer activity in a heterologous context rather than definitive evidence of erythroid-specific function. Moreover, K562 cells, while widely used as a human erythroid model, are of leukemic origin and represent an incompletely matured erythroid state, which may limit the generalizability of some observations.

In conclusion, our study demonstrates that LDB1 contributes to the regulation of fetal globin gene silencing during erythropoiesis, thereby enhancing our understanding of hemoglobin switching. These findings suggest that LDB1 functions as an important upstream component of globin regulation, potentially promoting adult globin gene expression while limiting fetal globin transcription. Also, our results open potential avenues for treating hemoglobinopathies through targeted gene regulation and underscore LDB1's importance in maintaining cellular function by preventing oxidative damage and supporting cell cycle progression.

## CRediT authorship contribution statement

**Si-Won Park:** Data curation, Formal analysis, Investigation, Methodology, Validation, Writing – original draft, Writing – review & editing. **Chang-Yong Choi:** Investigation, Methodology, Validation, Writing – original draft, Writing – review & editing. **In-Byung Park:** Data curation, Formal analysis, Investigation, Methodology, Validation, Writing – original draft, Writing – review & editing. **Seok-Jin Kang:** Investigation, Methodology, Writing – review & editing. **Hyeon Jeong Lee:** Methodology, Writing – review & editing. **Ji-In Kim:** Investigation, Writing – review & editing. **Joonbeom Bae:** Formal analysis, Investigation, Writing – original draft, Writing – review & editing. **Dongho Geum:** Formal analysis, Methodology, Writing – original draft, Writing – review & editing. **Yong-Pil Cheon:** Formal analysis, Writing – original draft, Writing – review & editing. **Taehoon Chun:** Conceptualization, Formal analysis, Funding acquisition, Supervision, Validation, Writing – original draft, Writing – review & editing.

## Declaration of competing interest

The authors declare that they have no known competing financial interests or personal relationships that could have appeared to influence the work reported in this paper.

## Data Availability

The raw sequencing data were deposited into the Gene Expression Omnibus (GEO) of the National Center for Biotechnology Information (NCBI) (https://www.ncbi.nlm.nih.gov/geo/) under the accession number GSE247801. The authors declare that all relevant data are included in the article and supplemental materials, and all materials described in this article can be accessed upon reasonable request.
