# Supplementary material for: LDB1 represses fetal hemoglobin expression by enhancing BCL11A transcription
Source: Redox Biol. 2026 Feb 4;91:104070. doi: 10.1016/j.redox.2026.104070 (PMC12907011; doi:10.1016/j.redox.2026.104070)
Supplement: Multimedia component 1 [file mmc1.pdf]

Supplementary material

LDB1 represses fetal hemoglobin expression by enhancing *BCL11A* transcription

Park *et al*.

Table S1. Primer sequences used in this study.

Genotyping PCR	Forward Primer	Reverse Primer
Ldb1	5'-CTTATGTGACCACAGCCATGCATGCATGTG-3'	5'-CAGCAAACGGAGGAAACGGAAGATGTCAG-3'
Vav1-Cre	5'-ATGGTGCCCAAGAAGAAGAG-3'	5'-CAGGTGCTGTTGGATGGTCT-3'
**qRT-PCR**	**Forward Primer**	**Reverse Primer**
Ldb1	5'-GTGACAATCTCTGGTGGGA-3'	5'-GGAAGTAGCGTGGTATCAG-3'
Hbb-bh1	5'-GGCTCTGCAGCTCTTCCAGAG-3'	5'-GAGCAAAGGTCTCCTTGAGGT-3'
Hbb-y	5'-TGGCCTGTGGAGTAAGGTCAA-3'	5'-GAAGCAGAGGACAAGTTCCCA-3'
Hbb-b1	5'-GACCCAGCGGTACTTTGATAGC-3'	5'-TGAGGCTGTCCAAGTGATTCA-3'
Bcl11a	5'-GTCTGCACACGGAGCTCTAA-3'	5'-CACTGGTGAATGGCTGTTTG-3'
Cbfa2t3	5'-CTTAAGCGTTTCCTCACCACC-3’	5’-CGTCAGAGTTGAGTTCACCAG-3’
Sox6	5'-GAGCTGCCTACACTTGTCA-3’	5’-GAGGTAGACGTATTTCGG-3’
Alas2	5'-CCATCTTAAGGCAACCAAGGC-3'	5'-ACAGCATGAAAGGACAATGGC-3'
Hmox1	5'-GCCGAGAATGCTGAGTTCATG-3'	5'-GCCGAGAATGCTGAGTTCATG-3'
Gsta3	5'-CCTGGCAAGGTTACGAAGT-3'	5'-GTCTGCACCAGTTTCATCC-3'
Hdc	5'-CGTTGCCTACACCTCTGA-3'	5'-CCTGTTGCTTGTCTTCCTC-3'
Aqp1	5'-GGGCAGTAATATCACTGTCTT-3'	5'-GTGGGTCCCTCACTTTCA-3'
Aqp3	5'-CTGGGGACCCTCATCCTT-3'	5'-TGGTGAGGAAGCCACCAT-3'
Aqp9	5'-CTCAACTCTGGTTGTGCCATGAA-3'	5'-ATCATAGGGCCCACGACAGGTA -3'
Cdkn1a	5'-GATCCACAGCGATATCCAGAC-3'	5'-CGAAGAGACAACGGCACACT-3'
Cdkn1b	5'-CAGACGTAAACAGCTCCGA-3'	5'-CCATTCAATGGAGTCAGCGA-3'
Cdkn1c	5'-AGAGAACTGCGCAGGAGAAC-3'	5'-TCTGGCCGTTAGCCTCTAAA-3'
Cdkn2b	5'-TCTGCAGCTGGATCTGGTC-3'	5'-ACCGCGTCCTGAAAGGTAG-3'
Cdkn2c	5'-CCTGTGGTGGAGTTCCTTA-3'	5'-CATAGAACCTGGCCAAGTCG-3'
Cdkn2d	5'-CCTGAAGGTTCTGGTGGAGCA-3'	5'-CTGTGGTGGAGATCAGATTCAGGA-3'
Ccng2	5'-ACCTCCACAGCAGCTACTAC-3'	5'-CCTCTCCACAACTCATGTCT-3'
Ccne1	5'-GACATAGACATTCAGCCAGGACACA-3'	5'-TCCAAAGTTGCACCAGTTTGCTTA-3'
Gapdh	5'-ATGGTGAAGGTCGGTGTGAA-3'	5'-GGTCGTTGATGGCAACAATCTC-3'
HBG	5'-TGGCAAGAAGGTGCTGACTTC-3'	5'-TCACTCAGCTGGGCAAAGG-3'
GAPDH	5'-ATGACCACAGTCCATGCCA-3'	5'-CCAGTAGAGGCAGGGATGA-3'
**ChIP-qPCR**	**Forward Primer**	**Reverse Primer**
Bcl11a #1	5'-CAGTGAGGATGGATGGAGT-3'	5'-CCTTATCTCACACCTACAGC-3'
Bcl11a #2	5'-GCAGCTTCCTTGATGGTCC-3'	5'-CTAGAAAGACAGCTCAGGCA-3'
Bcl11a #3	5'-TGGCTTTGGAGCACACTGA-3'	5'-CCACAGCGATAACCAGAG G-3'
Bcl11a #4	5'-GTGTGGATGGAGTGGCTGTTG-3'	5'-AAGCGGCGAGTGGCAGTTA-3'
Cbfa2t3 #1	5'-CGAGAGTCATGCCTTGTA-3’	5’-GGCCTGAGTTTATCTTCC-3’
Cbfa2t3 #2	5'-GATACCTAAGAGACCTGCTGG-3’	5’-GGAGTTATGGTACCTACTGG-3’
Cbfa2t3 #3	5'-ACTGCAGACGGCCATGGTT-3’	5’-GTAGACGTTGGTTGTGGCTT-3’
Cbfa2t3 #4	5'-TGGCCAGGATGGACATTACTC-3’	5’-CATGCCATTCCGGTCTGA-3’
Cbfa2t3 #5	5'-GTGAGTGTCTGTGGAACTGC-3’	5’-TGGTGACTGGAGCTTCTCT-3’
Cbfa2t3 #6	5'-CCAAGTCTTGCCAAGGAAGG-3’	5’-CCATGCATACAGTGAAGAGAGC-3’
Cbfa2t3 #7	5'-CAGGCAAACGTAGAGACTGG-3’	5’-GGCAACTCCGTAGACTACC-3’
Sox6 #1	5’-CTGTCTAAGGCAGGAAGC-3’	5’-GCGGCCTATTCATTATGG-3’
Sox6 #2	5’-TCTGCCTGGAACTTGCTG-3’	5’-GACCTTGCTATACCTCTCTG-3’
Sox6 #3	5’-CCTCCTCTGTGGTGTGAATGT-3’	5’-GAAATGACTGTCTGCCTCC-3’
Sox6 #4	5’-GCTGGAGAACATCTGAGACT-3’	5’-GTACATGGCAGAACTCTGGA-3’
Sox6 #5	5’-CTACCAATGTGGCCATACC-3’	5’-TGACAGCAGCCACTTCC-3’
Alas2 #1	5’-TACAGATTGCCAGAGCCAGC-3’	5’-GCTGAGCCTGCAGACCATA-3’
BCL11A #1	5’-CATCTTGTGTGCTTGGTCG-3’	5’-CAACAGTGATAACCAGCAGG-3’
BCL11A #2	5’-GCTGATTCCAGTGCAAAGTC-3’	5’-CTTCCTGGAGCCTGTGAT-3’
BCL11A #3	5’-GAGTTGCACAACCACGTAGT-3’	5’-CCTGGAGAAGAGCAAATG-3’
**Luciferase assay**	**Forward Primer**	**Reverse Primer**
Bcl11a intron #3	5’-CGGGATCCGGGGTCGAGGTTAGG-3’	5’-ACGCGTCGACTCTGAGAAAGATGAG-3’
Bcl11a intron #4	5’-CGGGATCCCTCCTACAAGTCAGA-3’	5’-ACGCGTCGACCGTAATTTTAACCTT-3’
Ldb1	5’-GCGAATTCATGTCAGTGGGCTGTGCCT-3’	5’-TAGGATCCTCACTGGGAAGCCTGTGACG-3’
Lmo2	5’-TATCTAGAATGGAAGGGAGCGCGGTGACT-3’	5’-GCGAATTCCTAGATGATCCCATTGATCT-3’
Gata1	5’-GCTCTAGAATGGATTTTCCTGGT-3’	5’-CGGGATCCTCAAGAACTGAGTGG-3’
**Overexpression of *Bcl11a***	**Forward Primer**	**Reverse Primer**
Bcl11a	5’-CGGGATCCATGTCTCGCCGCAAG-3’	5’-ATAAGAATGCGGCCGCTCAGAACTTAAGGGT-3’

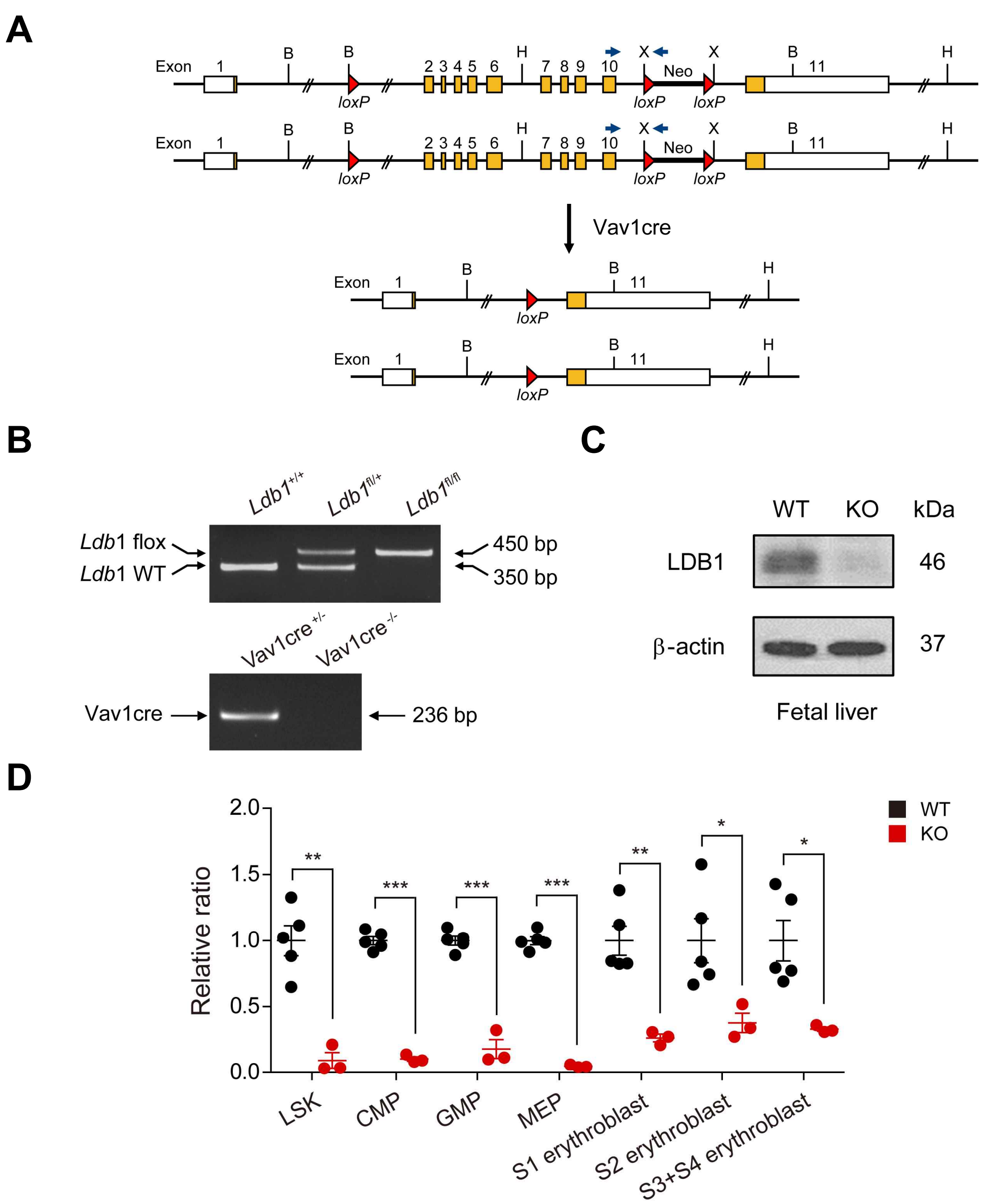

Fig. S1. Generation of mice with hematopoietic cell-specific *Ldb1* deletion. (**A**) A schematic representation of the Cre-mediated homologous recombination of the *Ldb1* allele. The floxed *Ldb1* allele was generated by inserting a *loxP* site between exon 1 and exon 2, and a *loxP*-Neo-*loxP* cassette into the tenth intron of the *Ldb1* locus. Red triangles indicate the *loxP* sites, the orange boxes represent the coding DNA sequences, and the white boxes indicate the 5' and 3' untranslated regions of the *Ldb1* locus. The blue arrows indicate the forward and reverse primers used for genotyping the *Ldb1* allele. B, *Bam*HI site; H, *Hind*III site, X, *Xba*I site; Neo, neomycin resistance gene. (**B**) Genotyping PCR was performed using genomic DNA isolated from the tails of *Ldb1*$^{+/+}$, *Ldb1*$^{fl/+}$, *Ldb1*$^{fl/fl}$, Vav1cre$^{+/-}$, and Vav1cre$^{-/-}$ mice. (**C**) LDB1 expression levels in E14.5 FLs of the *Ldb1*$^{fl/fl}$Vav1cre^{-} (WT) and *Ldb1*$^{fl/fl}$Vav1cre^{+} (KO) mice were determined by Western blot analysis. $n = 3$. (**D**) *Ldb1* mRNA expression levels in hematopoietic lineages from E14.5 WT ($n = 5$) and KO ($n = 3$) FLs were measured by qRT-PCR. LSK, Lin^{-}Sca-1^{+}c-Kit^{+} cells; CMP, common myeloid progenitor; GMP, granulocyte/monocyte progenitor; MEP, megakaryocyte/erythrocyte progenitor. Statistical significance was evaluated with a two-tailed Student's *t*-test. *$P < 0.05$; **$P < 0.01$; ***$P < 0.001$. All data are presented as means ± SEM.

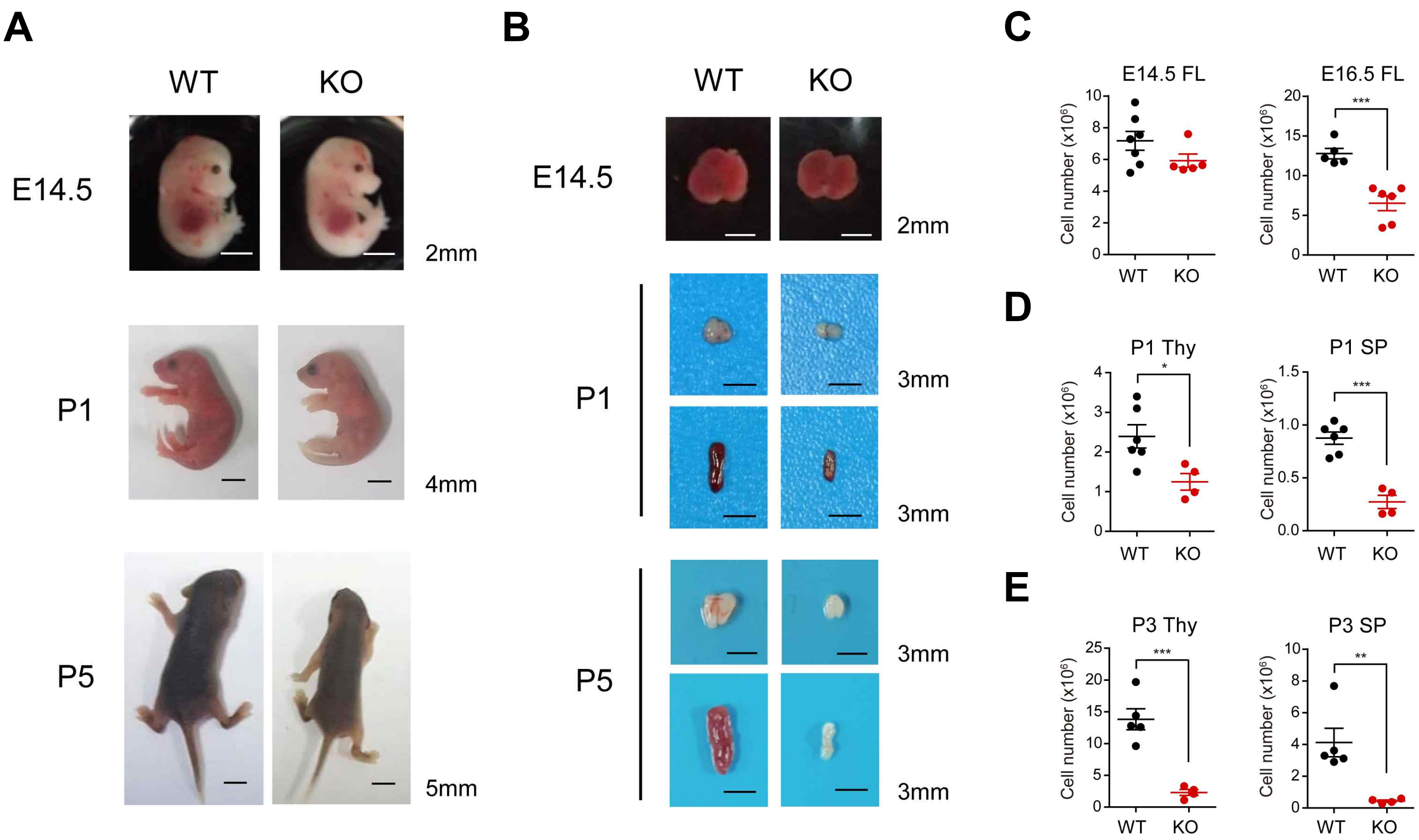

Fig. S2. Hematopoietic cell-specific *Ldb1* deficiency in mice leads to severe hypoplasia of the thymus and spleen, and causes early lethality shortly after birth due to defective erythropoiesis. (**A**) Appearance of E14.5 fetuses, and P1 and P5 pups in WT and KO mice. (**B**) Representative images showing the appearance of E14.5 fetuses, and P1 and P5 pups in WT and KO mice. (**C**) Absolute numbers of E14.5 WT (n = 7) and KO (n = 5) FL cells (FLCs) as well as E16.5 WT (n = 5) and KO (n = 6) FLCs. (**D**) Absolute cell numbers of P1 thymi (P1 Thy) and spleens (P1 SP) from WT (n = 6) and KO (n = 4) mice. (**E**) Absolute cell numbers of P3 thymi (P3 Thy) and spleens (P3 SP) from WT (n = 5) and KO (n = 4) mice. Statistical significance was evaluated with a two-tailed Student's t-test. *$P < 0.05$; **$P < 0.01$; ***$P < 0.001$. All data are presented as means ± SEM.

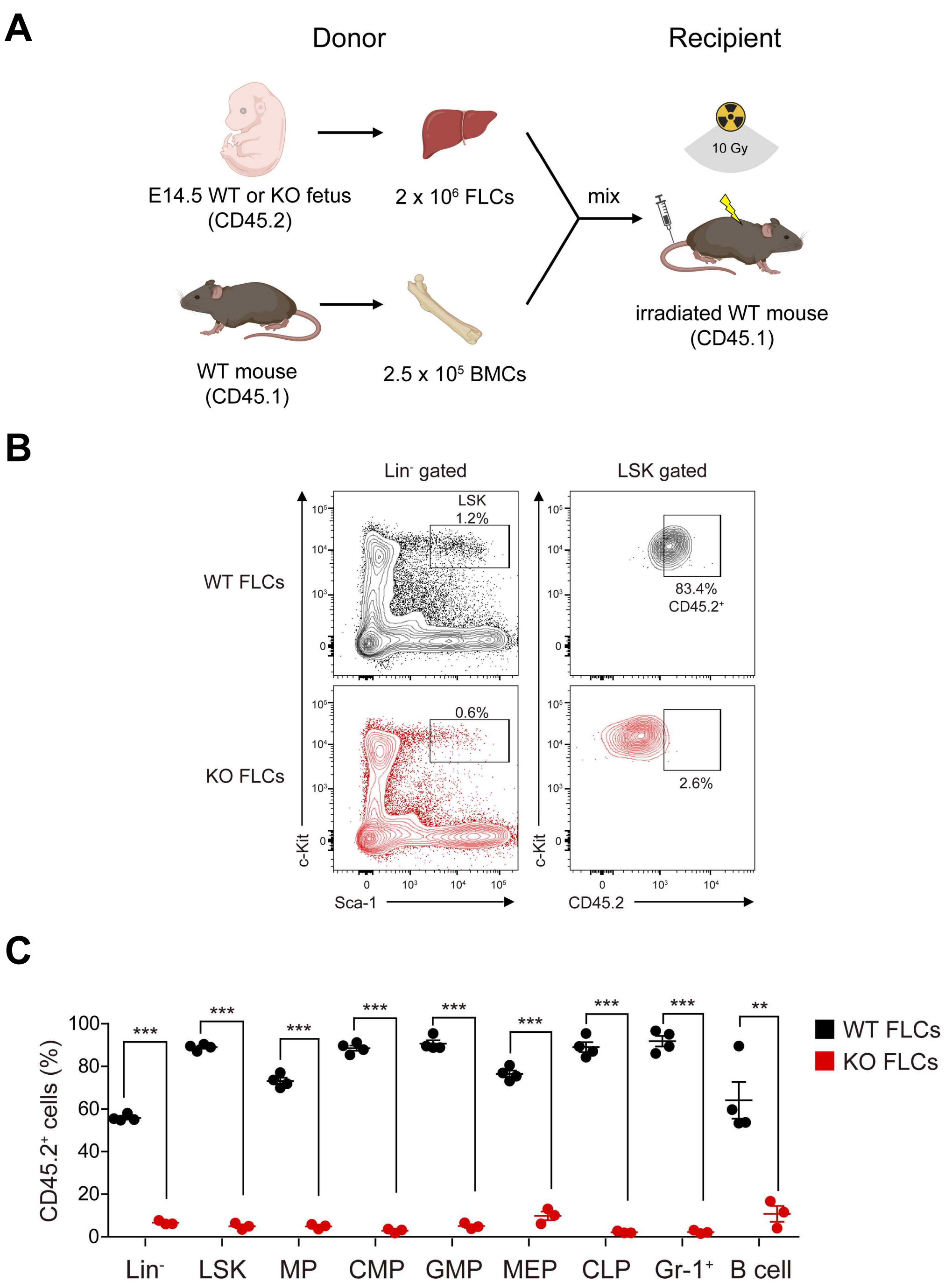

Fig. S3. Hematopoietic cell-specific *Ldb1* deletion results in impaired maintenance of the proper function of long-term hematopoietic stem cells (LT-HSCs). (**A**) Schematic diagram of competitive repopulation assays. The diagram was created with BioRender.com. Lethally irradiated WT CD45.1 mice were transplanted with E14.5 FLCs (2×10^6 cells) from WT or KO fetuses (CD45.2) and BMCs (2.5×10^5 cells) from WT CD45.1 mice. Twelve weeks post-transplantation, recipient BMCs were analyzed for the reconstitution of donor FLC-derived immune cells (CD45.2). (**B**) Representative flow cytometry plots depicting the frequency of donor FLC-derived LSKs (CD45.2) in the BM of recipients (CD45.1). WT FLCs, E14.5 WT FLCs (CD45.2) + WT BMCs (CD45.1); KO FLCs, E14.5 KO FLCs (CD45.2) + WT BMCs (CD45.1). (**C**) Frequency of HSPCs and immune cell populations (CD45.2) derived from WT FLCs ($n = 4$) or KO FLCs ($n = 3$) in the BM of recipients (CD45.1). CLP, common lymphoid progenitor; Gr-1$^+$, granulocytes. Statistical significance was assessed by two-tailed Student's *t*-test. $^{**}P < 0.01$; $^{***}P < 0.001$. All data are presented as the mean ± SEM.

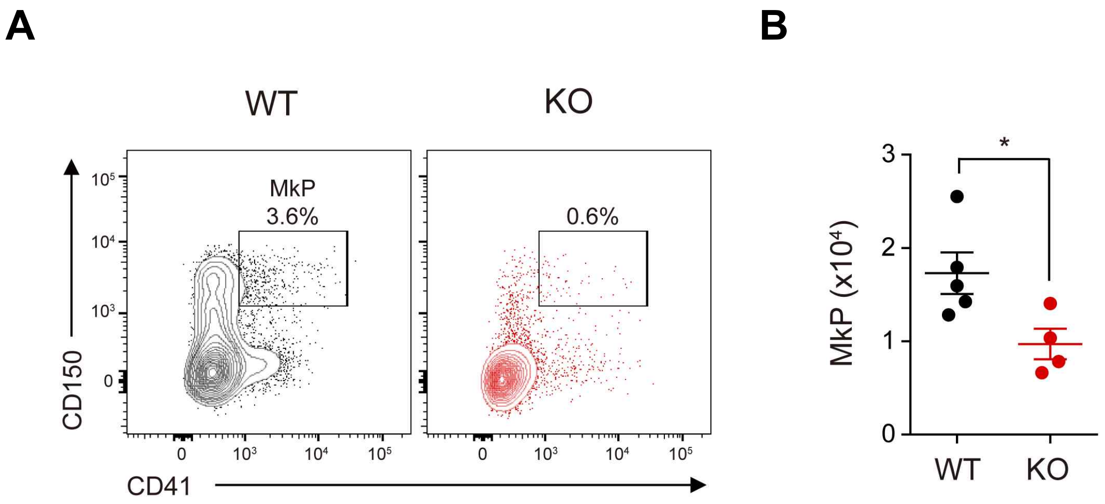

Fig. S4. Hematopoietic cell-specific *Ldb1* deficiency leads to a reduction in the number of megakaryocyte precursors. Representative flow cytometry plots (**A**) and absolute numbers (**B**) of megakaryocyte precursors (MkP) from E14.5 WT (n = 5) and KO (n = 4) FLs. Statistical significance was assessed by two-tailed Student's t-test. *$P < 0.05$. All data are presented as the mean ± SEM.

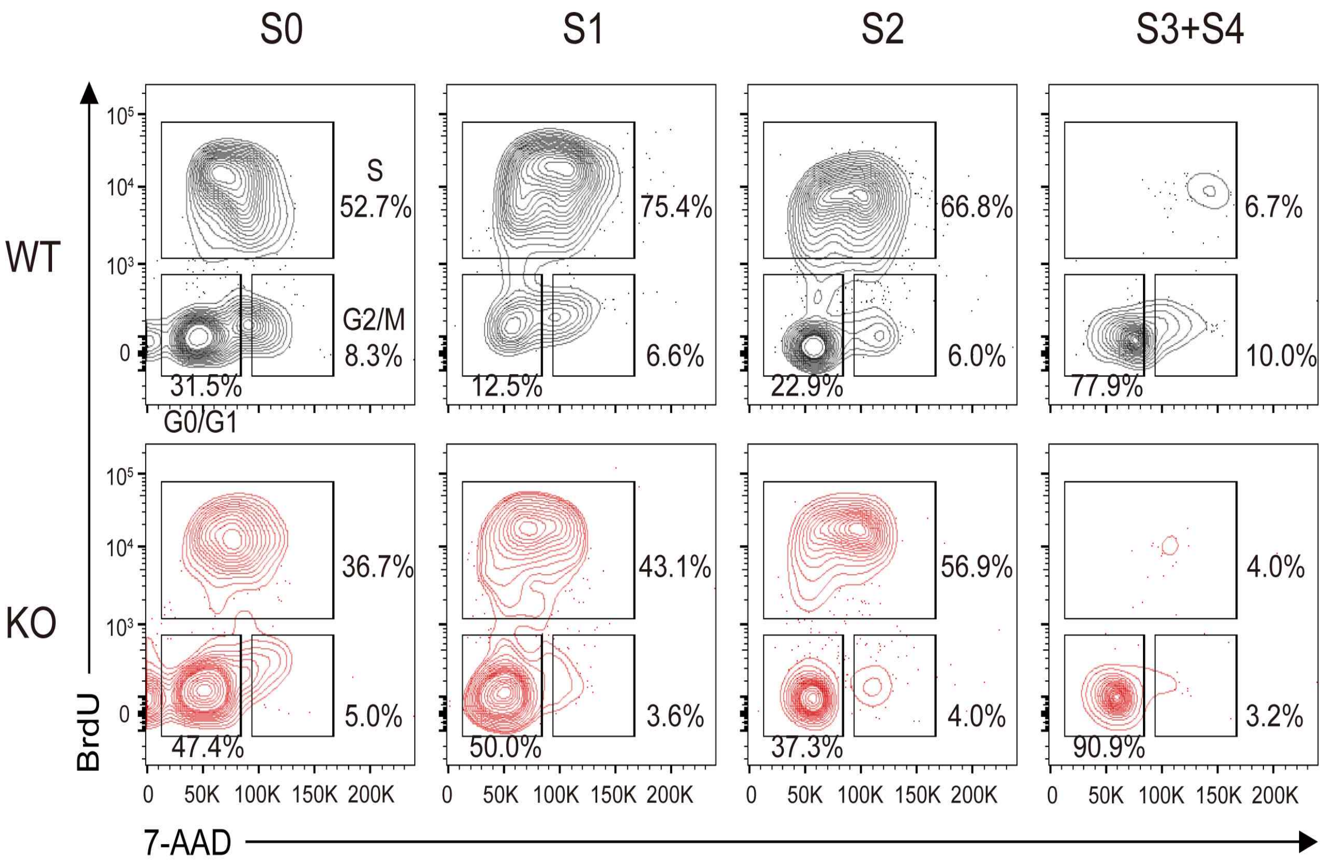

Fig. S5. *Ldb1* deficiency induces cell cycle arrest in proerythroblasts (S1 cells) from E14.5 FLCs. Representative flow cytometry plots analyzing the cell cycle phases of erythrocyte precursors at different stages (S0 to S4 cells) from WT (n = 6) and KO (n = 5) E14.5 FLCs.

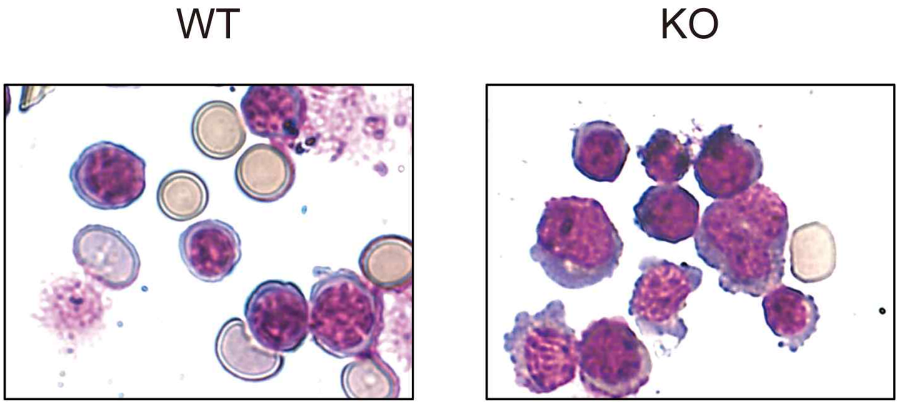

Fig. S6. *Ldb1* deficiency blocks erythrocyte maturation in E14.5 FL. Representative images of Giemsa-stained erythroid cells from E14.5 WT (*n* = 5) and KO (*n* = 5) FLs.

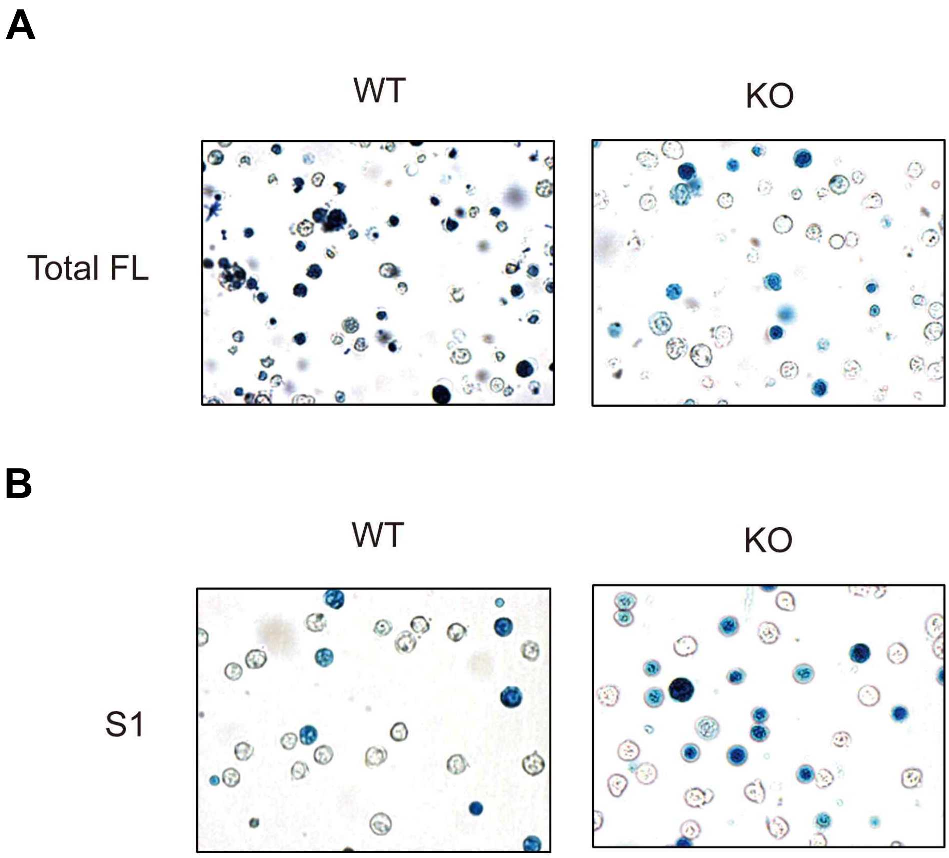

Fig. S7. ***Ldb1*** **deficiency reduces the absolute number of mature hemoglobinized cells but increases the number of hemoglobinized proerythroblasts (S1 cells) in the E14.5 FL.** Representative benzidine staining images of total FLCs (**A**) and proerythroblasts (S1 cells) (**B**) from E14.5 WT (n = 6) and KO (n = 6) fetuses.

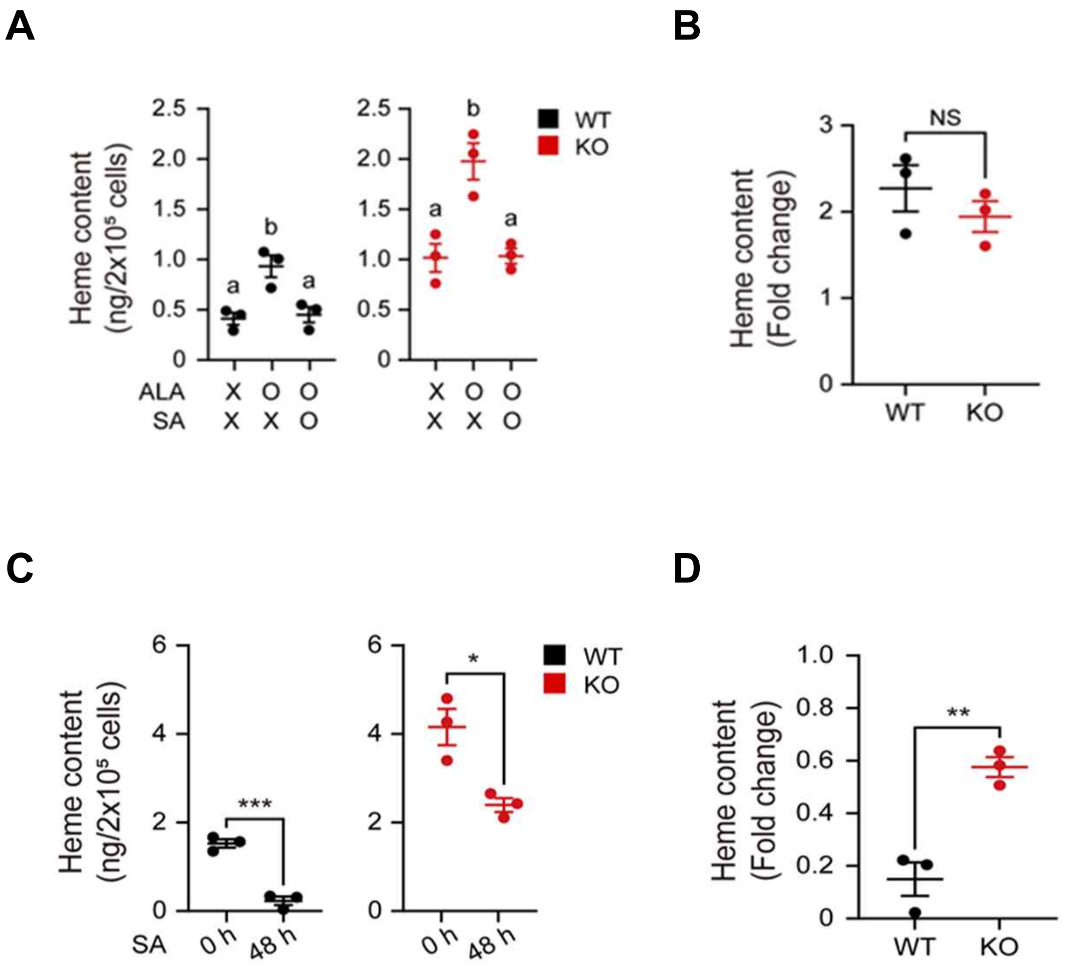

Fig. S8. *Ldb1* deficiency in erythrocyte precursors causes ROS accumulation due to impaired degradation of heme contents. (**A** and **B**) Heme synthesis is not altered in KO erythrocyte precursors compared with that in WT. (**A**) $Ter119^-$ cells (S0 and S1 cells) from E14.5 WT and KO FLs were cultured in the presence of 5-aminolevulinic acid (ALA, 0.5 mM) and/or succinylacetone (SA, 0.5 mM). After one hour of culture, cells were harvested and heme contents were measured. $n = 3$. Statistical significance was assessed using one-way ANOVA with Tukey HSD analysis. Mean values not sharing the same superscript letter ($^{a, b}$) differ significantly at $P < 0.05$. (**B**) After the assay, relative heme synthesis between WT and KO erythrocyte precursors was calculated. $n = 3$. Statistical significance was assessed by two-tailed Student's *t*-test. NS, not significant; X, no treatment; ◯, treatment. (**C** and **D**) Degradation of heme is impaired in KO erythrocyte precursors compared with that in WT. $n = 3$. Statistical significance was assessed by two-tailed Student's *t*-test. (**C**) $Ter119^-$ cells (S0 and S1 cells) from E14.5 WT and KO FLs were cultured in the presence of SA (0.5 mM). After 48 h of culture, cells were harvested and heme contents were measured. (**D**) After the assay, relative heme degradation between WT and KO erythrocyte precursors was calculated. *$P < 0.05$; **$P < 0.01$; ***$P < 0.001$. All data are presented as the mean ± SEM.

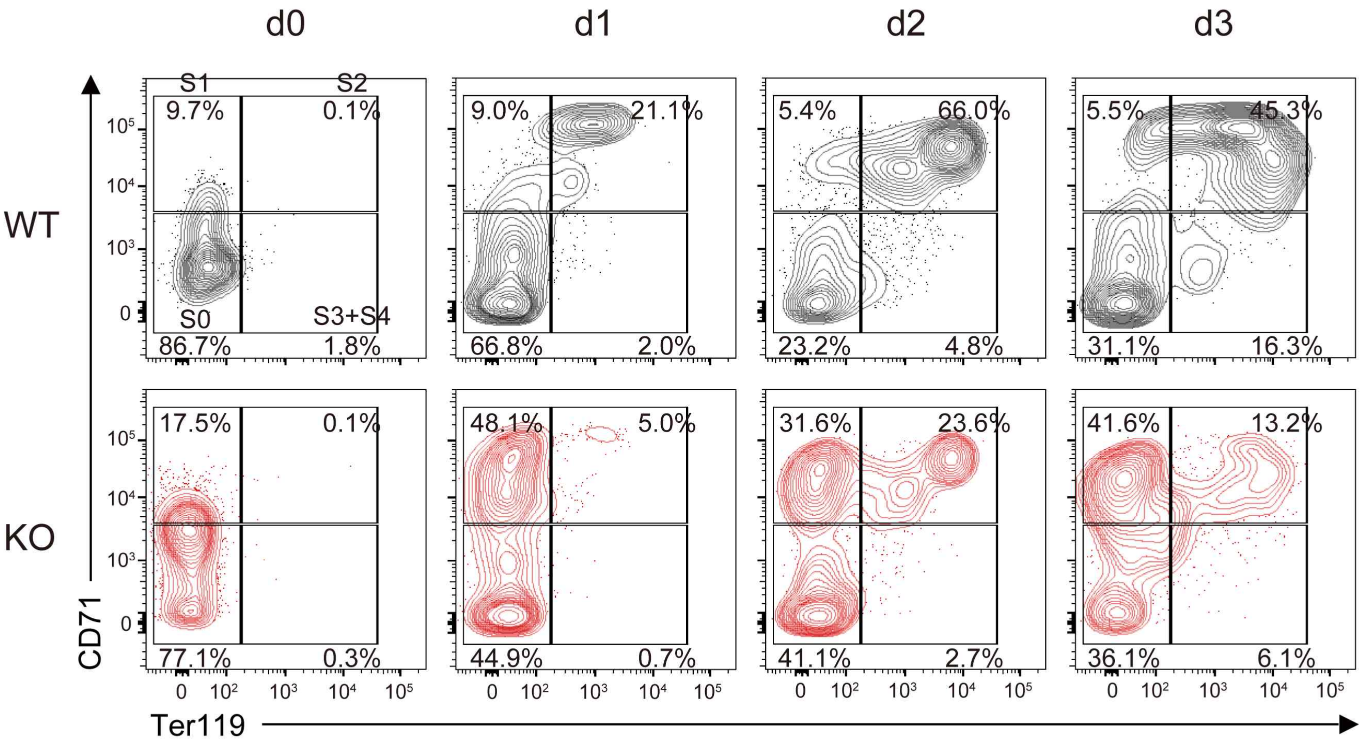

Fig. S9. Frequency of each stage of erythrocyte precursors from E14.5 WT and KO Ter119[-] cells during *in vitro* differentiation. Representative plots illustrate the percentage of cells at each precursor stage (S0 to S4 cells) in E14.5 WT (n = 5) and KO (n = 4) Ter119[-] cells during the culture. d, days after culture.

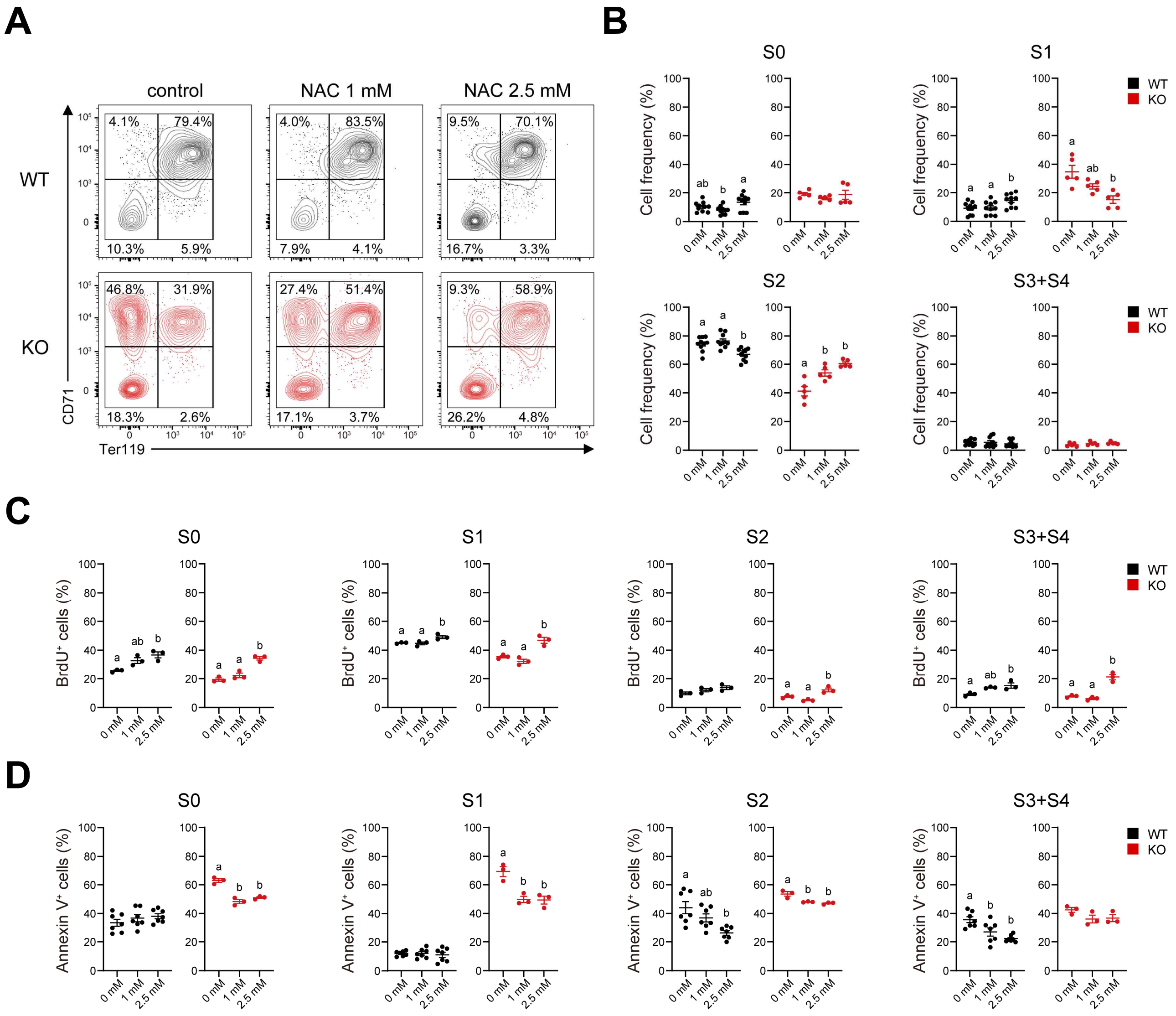

Fig. S10. KO erythrocyte precursors showed improved survival, proliferation, and maturation following treatment with the ROS scavenger. Ter119^{-} cells (S0 and S1 cells) from E14.5 WT and KO FLs were cultured for 3 days in the presence of 1 mM or 2.5 mM *N*-acetylcysteine (NAC). (**A** and **B**) Representative plots illustrating the percentage of cells at each precursor stage (S0 to S4 cells) (**A**) and frequency of erythrocyte precursors at each stage (S0 to S4 cells) (**B**) on day 3 after culture. WT, n = 10; KO, n = 5. (**C**) Frequency of BrdU^{+} cells at each stage of erythrocyte precursors on day 3 after culture. n = 3. (**D**) Frequency of Annexin V^{+} cells at each stage of erythrocyte precursors on day 3 of culture. WT, n = 7; KO, n = 3. Statistical significance was assessed using one-way ANOVA with Tukey HSD analysis. Mean values not sharing the same superscript letter ($^{a,\, b}$) differ significantly at $P < 0.05$. All data are presented as the mean ± SEM.

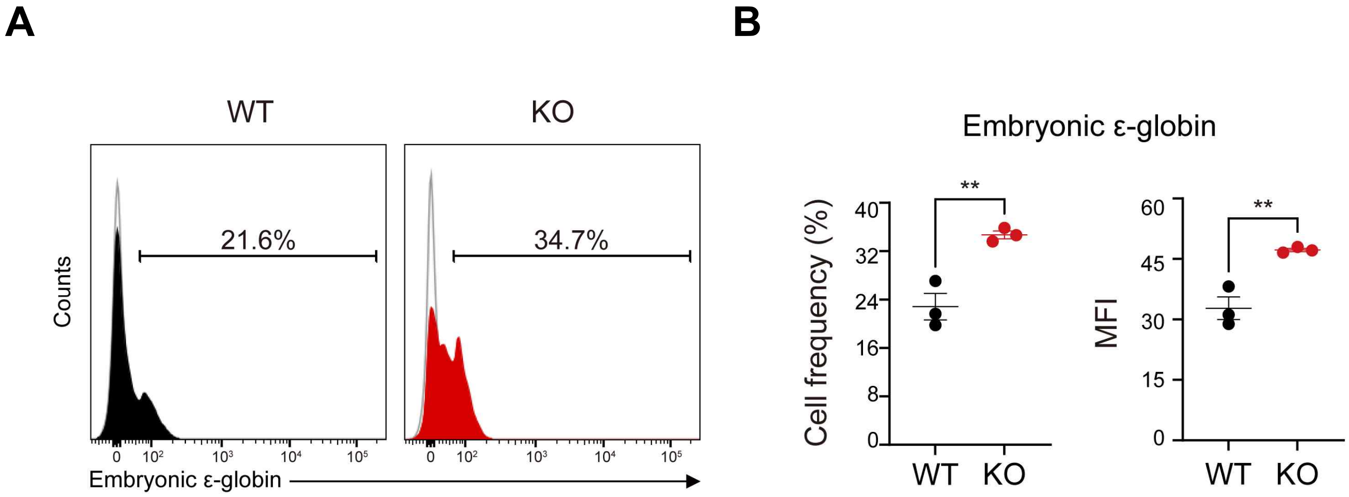

Fig. S11. Embryonic globin expression was significantly higher in KO S1 cells compared to WT S1 cells from E14.5 FLs. Representative flow cytometry plots (**A**) and cell frequency (*left*) and mean fluorescence intensity (MFI) (*right*) (**B**) of embryonic ε-globin (HBBY) expression in S1 cells from E14.5 WT and KO FLs. $n = 3$. The isotype control is shown as a gray line. Statistical significance was assessed by two-tailed Student's t-test. $^{**}P < 0.01$. All data are presented as the mean ± SEM.

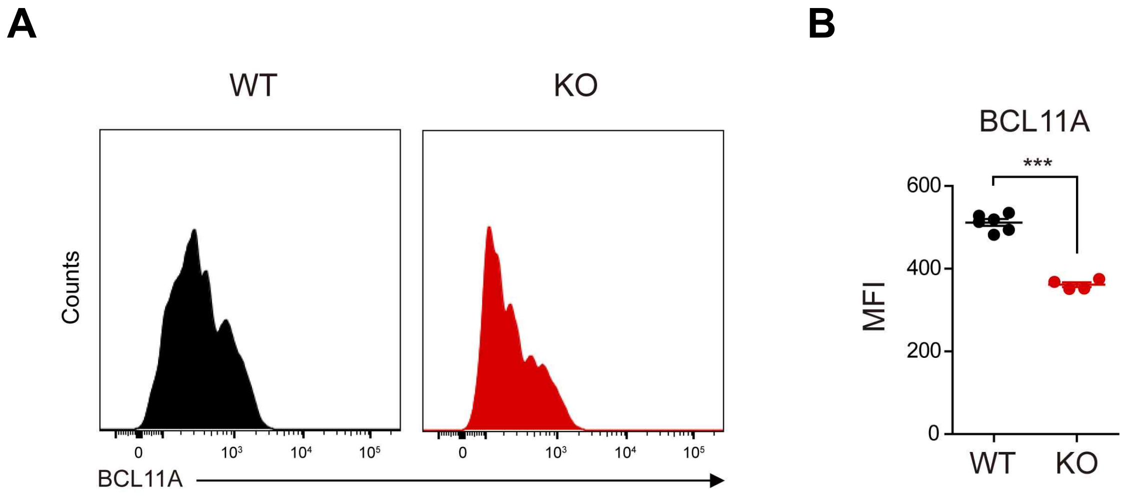

Fig. S12. Reduced BCL11A expression in KO S1 cells compared to WT S1 cells from E14.5 FLs. Representative flow cytometry plots (**A**) and mean fluorescence intensity (MFI) (**B**) of BCL11A expression in S1 cells from E14.5 WT ($n = 6$) and KO ($n = 4$) FLs. Statistical significance was assessed by two-tailed Student's *t*-test. ***$P < 0.001$. All data are presented as the mean ± SEM.

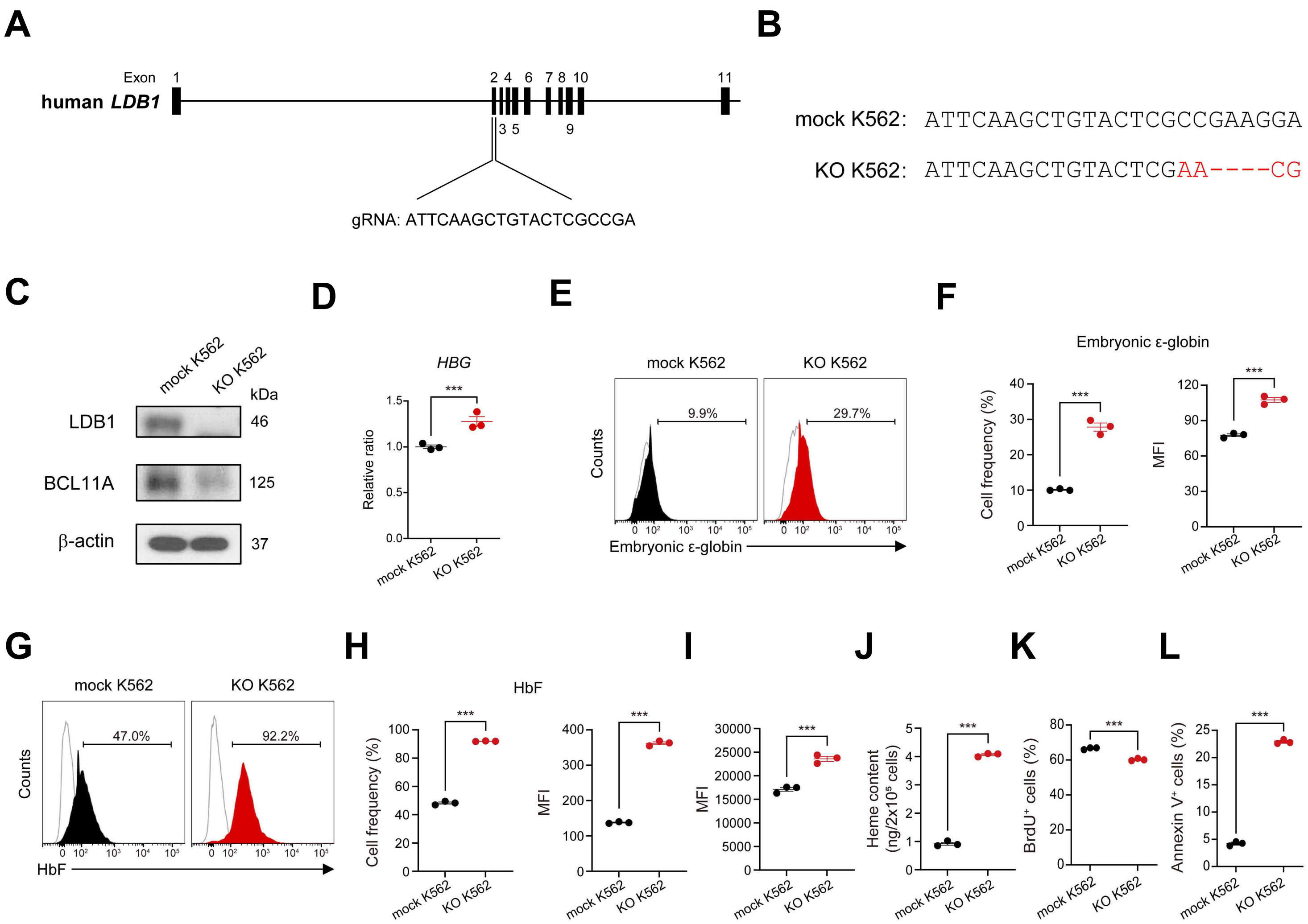

Fig. S13. LDB1 deficiency in K562 cells causes decreased cell proliferation and increased apoptosis, accompanied by elevated embryonic and fetal globin expression and ROS accumulation, similar to the phenotype observed in S1 cells from KO E14.5 FLs. (**A**) Designing guide RNA (gRNA) to target exon 2 of the human *LDB1* gene. (**B**) Genomic DNA sequence alignment of the *LDB1* knockout K562 cells (KO K562) compared to the empty vector-transfected K562 cells (mock K562). (**C**) Representative immunoblot images showing *LDB1* and *BCL11A* expression in mock K562 and KO K562. $n = 3$. (**D**) mRNA expression levels of human fetal globin γ (*HBG*) in mock K562 and KO K562, measured by qRT-PCR analysis. $n = 3$. (**E** and **F**) Representative flow cytometry plots (**E**) and cell frequency (*left*) and mean fluorescence intensity (MFI) (*right*) (**F**) of embryonic ε-globin (HBE1) expression in mock K562 and KO K562. $n = 3$. The isotype control is shown as a gray line. (**G** and **H**) Representative flow cytometry plots (**G**) and cell frequency (*left*) and MFI (*right*) (**H**) of fetal hemoglobin (HbF) expression in mock K562 and KO K562. $n = 3$. The isotype control is shown as a gray line. (**I**) MFI after staining with CellROX in mock K562 and KO K562. $n = 3$. (**J**) Cellular heme content in mock K562 and KO K562. $n = 3$. (**K**) Frequency of $BrdU^+$ cells in mock K562 and KO K562. $n = 3$. (**L**) Frequency of Annexin V^+ cells in mock K562 and KO K562. $n = 3$. Statistical significance was assessed by two-tailed Student's *t*-test. $^{***}P < 0.001$. All data are presented as the mean ± SEM.

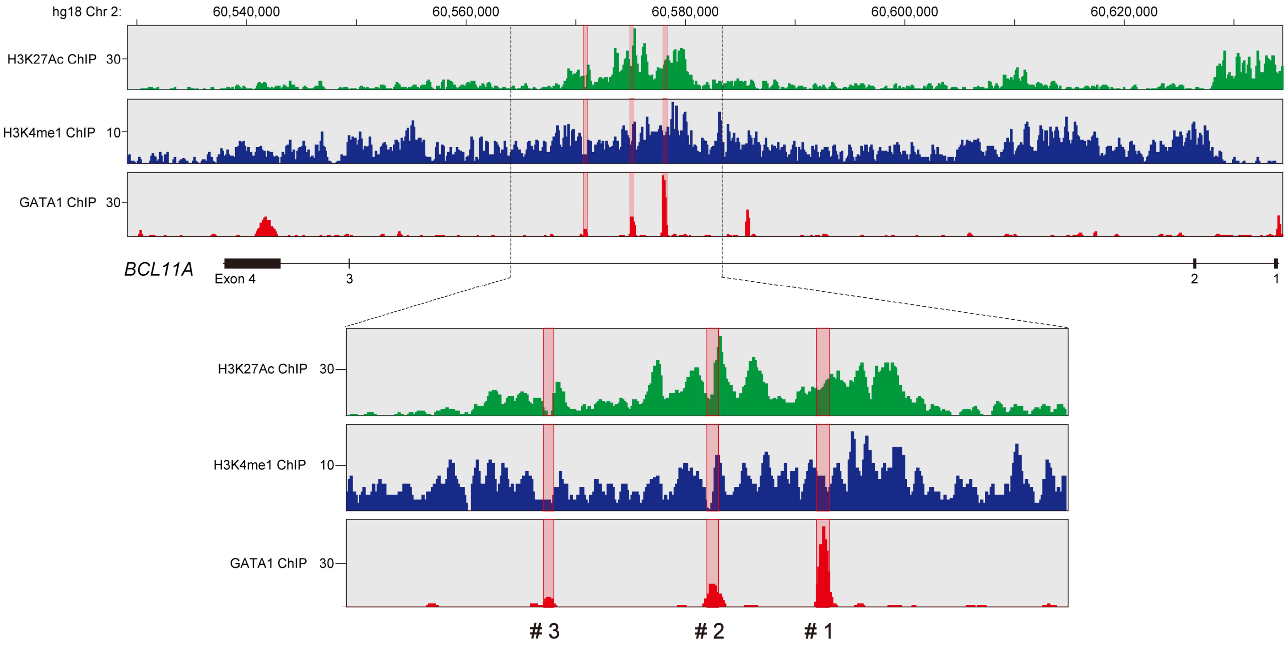

Fig. S14. Occupancy of H3K27Ac, H3K4me1, and GATA1 at the *BCL11A* locus in primary human erythroblasts (GEO accession: GSE36994). Three primer sets, labeled as #1, #2, and #3, for ChIP-qPCR assays were designed within intron 2 of the human *BCL11A* locus.

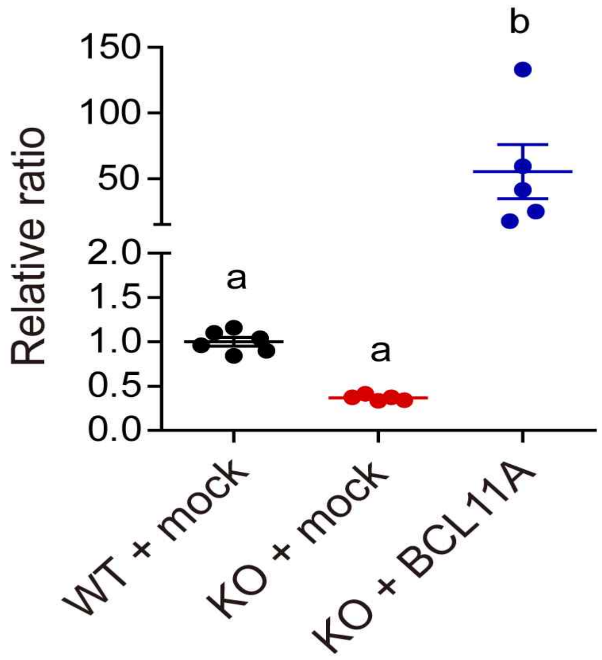

Fig. S15. Ectopic expression of *Bcl11a* in erythrocyte precursors from E14.5 WT and KO FLs. Ter119^{-} cells from E14.5 WT and KO FLs were transduced with either an empty vector (mock) or a *Bcl11a*-expressing vector (BCL11A). Eighteen h after transduction, *Bcl11a* mRNA expression levels were monitored via qRT-PCR. WT + mock (n = 6), Empty vector-transduced Ter119^{-} cells from E14.5 WT FLs; KO + mock (n = 5), Empty vector-transduced Ter119^{-} cells from E14.5 KO FLs; KO + BCL11A (n = 5), *Bcl11a*-expressing vector-transduced Ter119^{-} cells from E14.5 KO FLs. Statistical significance was assessed using one-way ANOVA. Mean values not sharing the same superscript letter ([a, b]) differ significantly at $P < 0.05$. All data are presented as the mean ± SEM.

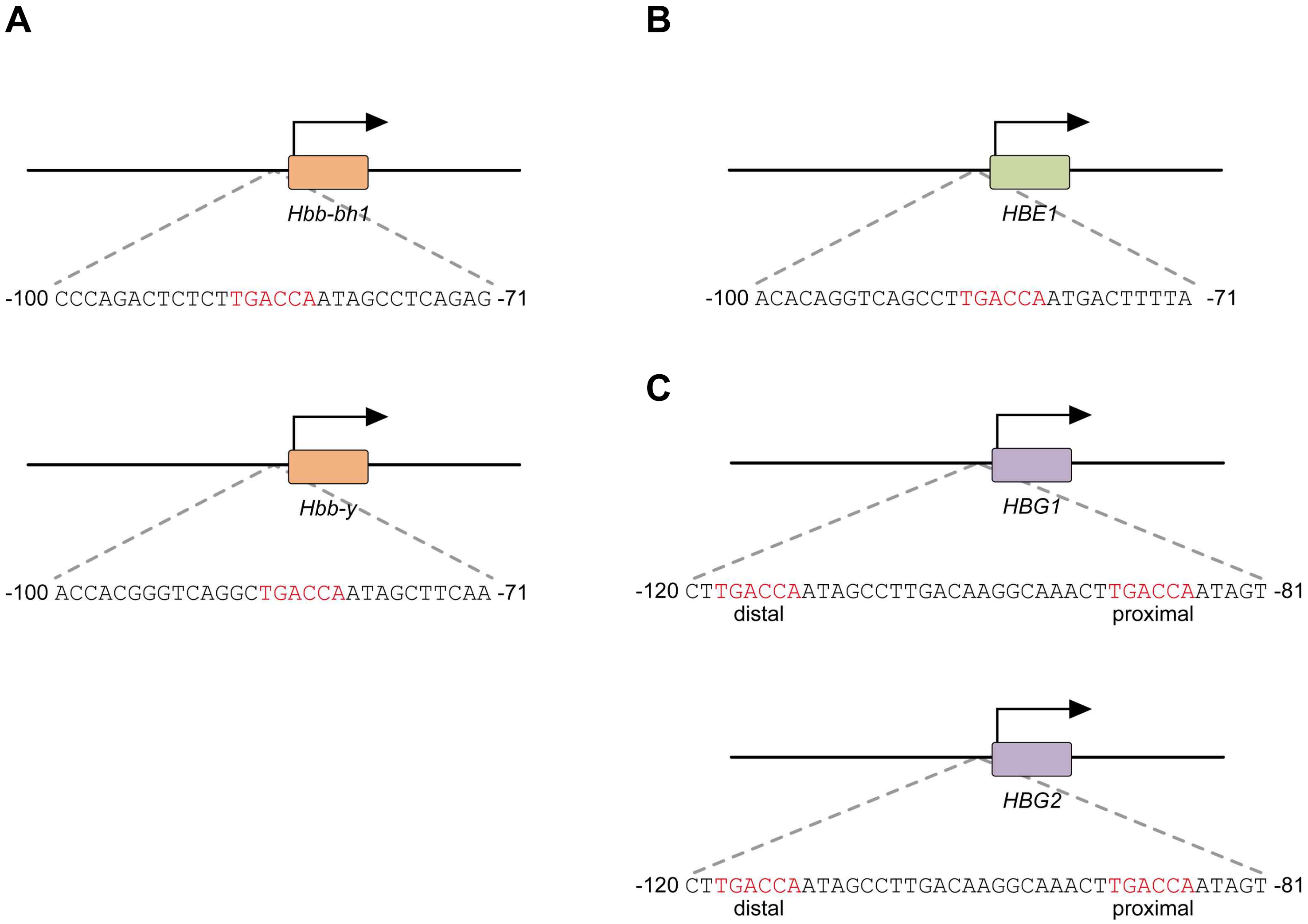

Fig. S16. The putative BCL11A binding motifs are found in embryonic and fetal globin promoters of humans and mice. (**A**) The putative BCL11A binding motifs at mouse embryonic β-globin promoters (*Hbb-y* and *Hbb-bh1*). (**B**) The putative BCL11A binding motifs at human embryonic (*HBE1*) and fetal globin promoters (*HBG1* and *HBG2*). Nucleotide sequences indicating the putative BCL11A binding motif (TGACCA) are shown in red [47, 52].
